# Disrupting MLV integrase:BET protein interaction biases integration into quiescent chromatin and delays but does not eliminate tumor activation in a *MYC/Runx2* mouse model

**DOI:** 10.1371/journal.ppat.1008154

**Published:** 2019-12-09

**Authors:** Lorenz Loyola, Vasudevan Achuthan, Kathryn Gilroy, Gillian Borland, Anna Kilbey, Nancy Mackay, Margaret Bell, Jodie Hay, Sriram Aiyer, Dylan Fingerman, Rodrigo A. Villanueva, Ewan Cameron, Christine A. Kozak, Alan N. Engelman, James Neil, Monica J. Roth

**Affiliations:** 1 Rutgers-Robert Wood Johnson Medical School, Dept of Pharmacology, Piscataway, New Jersey, United States of America; 2 Department of Cancer Immunology and Virology, Dana-Farber Cancer Institute, Boston, Massachusetts, United States of America; 3 Harvard Medical School, Department of Medicine, Boston, Massachusetts, United States of America; 4 Beatson Institute for Cancer Research, Institute of Cancer Sciences, University of Glasgow, Glasgow, United Kingdom; 5 MRC Univ. of Glasgow Centre for Virus Research, College of Medicine, Veterinary Medicine and Life Sciences, University of Glasgow, Glasgow, United Kingdom; 6 Univ. of Glasgow School of Veterinary Medicine, Department of Veterinary Pathology Bearsden, United Kingdom; 7 NIH, NIAID, Bethesda, Maryland, United States of America; 8 Harvard Medical School, Department of Medicine, Boston, Massachusetts, United States of America; Duke University Medical Center, UNITED STATES

## Abstract

Murine leukemia virus (MLV) integrase (IN) lacking the C-terminal tail peptide (TP) loses its interaction with the host bromodomain and extraterminal (BET) proteins and displays decreased integration at promoter/enhancers and transcriptional start sites/CpG islands. MLV lacking the IN TP via an altered open reading frame was used to infect tumorigenesis mouse model (*MYC/Runx2)* animals to observe integration patterns and phenotypic effects, but viral passage resulted in the restoration of the IN TP through small deletions. Mice subsequently infected with an MLV IN lacking the TP coding sequence (TP^-^) showed an improved median survival by 15 days compared to wild type (WT) MLV infection. Recombination with polytropic endogenous retrovirus (ERV), *Pmv20*, was identified in seven mice displaying both fast and slow tumorigenesis, highlighting the strong selection within the mouse to maintain the full-length IN protein. Mapping the genomic locations of MLV in tumors from an infected mouse with no observed recombination with ERVs, TP^-^16, showed fewer integrations at TSS and CpG islands, compared to integrations observed in WT tumors. However, this mouse succumbed to the tumor in relatively rapid fashion (34 days). Analysis of the top copy number integrants in the TP^-^16 tumor revealed their proximity to known MLV common insertion site genes while maintaining the MLV IN TP^-^ genotype. Furthermore, integration mapping in K562 cells revealed an insertion preference of MLV IN TP^-^ within chromatin profile states associated with weakly transcribed heterochromatin with fewer integrations at histone marks associated with BET proteins (H3K4me1/2/3, and H3K27Ac). While MLV IN TP^-^ showed a decreased overall rate of tumorigenesis compared to WT virus in the *MYC/Runx2* model, MLV integration still occurred at regions associated with oncogenic driver genes independently from the influence of BET proteins, either stochastically or through trans-complementation by functional endogenous Gag-Pol protein.

## Author summary

Many different retroviruses, including murine leukemia virus (MLV), are used as vectors for human gene therapy and cancer immunotherapies (CAR-T) because of their stable and efficient delivery of genetic material into the host DNA. However, this process can result in activation and/or disruption of cellular genes, which has resulted in the outgrowth of tumors in previous clinical trials. Of critical importance is the preferred location within the host genome at which the retrovirus integrates. Our study presents a modified MLV virus that has lost the ability to bind to the host BET proteins, the drivers of insertion at promoter/enhancer regions of highly activated genes. This is the first direct study within an animal model to examine whether infection with this type of modified MLV virus affects tumor progression. We found that the modified virus improved the survival of the well-characterized *MYC/Runx2* mouse compared to infections with the wild-type MLV. Most modified viral integrants mapped into less active regions of the genome, but some integration events still occurred near cancer-related genes. Thus, while the modified MLV virus can be a safer vector than the wildtype virus, it still maintains the potential to activate oncogenes. This study provides new insights on how to improve the safety of MLV retroviral vectors.

## Introduction

Integration is an essential step for retroviral replication and pathogenesis (for review [1]). The integration substrate generated through reverse transcription is linear viral DNA containing a copy of the long terminal repeat (LTR) at each end. Integration is driven by the viral integrase (IN), structurally defined by distinct functional domains and regions, including the N-terminal region (NTR) containing the N-terminal extension (NED) and HHCC zinc-binding domain (NTD) [2], the catalytic core domain (CCD) [3], and the C-terminal domain (CTD) containing an unstructured tail peptide (TP) [4, 5]. Integration proceeds via two distinct IN catalytic activities; 3’ processing and strand transfer. During processing of the gammaretrovirus murine leukemia virus (MLV) LTR, a TT dinucleotide is hydrolyzed, exposing CA_OH_ 3’ ends. IN then uses the 3’ hydroxyl groups to cut the chromosomal DNA in a staggered fashion, which joins the viral DNA 3’ ends to the 5’ phosphates of the host DNA cut. Repair of the gapped integration intermediate, which contains unjoined viral DNA 5’ ends with protruding 5’-AA single stranded (ss) DNA tails, yields a 4 bp duplication of host chromosomal DNA flanking the integrated MLV provirus [6, 7].

Host protein interactions are key determinants of integration preferences of retroviruses [8–11]. MLV displays preferential integration near regions of high transcriptional activity, such as promoter and enhancer regions and transcription start sites (TSSs) [12, 13]. The interaction of the MLV IN with the host bromo- and extraterminal (BET) domain proteins influences target-site selection. BET proteins interact with various chromatin remodeler proteins [14], therefore guiding MLV integration to highly transcriptionally active chromatin regions. MLV IN has an unstructured TP within the CTD that interacts with the extraterminal (ET) domain of BET proteins [4, 5]. Removal of this interaction by substitution or truncation of the TP reduces preferential integration at TSS and CpG islands, which can redistribute the integration profile and decrease the oncogenic effects of MLV insertional mutagenesis [4, 15].

In mice, Moloney MLV (M-MLV) is a non-acute retrovirus and thus insertional activation of proto-oncogenes at identified common insertions sites (CISs) is the predominant mechanism of oncogenesis [1, 16], requiring a long-latency period varying between 4–12 months [16]. A transgenic mouse overexpressing two MLV CIS genes, *MYC* and *Runx2*, from CD2 promoters exhibits early onset lymphomagenesis through a synergistic mechanism that is proposed to neutralize p53 activation [17, 18]. Infection of *MYC/Runx2* mice with WT MLV reduced animal survival by 10 days [19–21]. Additionally, neonatal infection of this mouse model with M-MLV WT virus accelerated tumorigenesis and increased clonal complexity through various insertional mutagenesis sites [20]. Analysis of these integration sites through next-generation sequencing and subsequent comparison with reference genomes and ChIP-seq data sets identified a panel of MLV CIS that accelerated the oncogenic process [20]. *MYC/Runx2* mice are accordingly an established model to study the relationship between M-MLV integration at predetermined CISs and tumorigenesis [20, 21].

Murine gammaretroviruses are classified based on their exogenous versus endogenous localization and receptor usage [22, 23]. Inbred strains of mice harbor endogenous type C MLVs, which fall into three general classes depending on their receptor usage, and thus their host and tissue specificities. These classes include the ecotropic viruses, limited to rodents (mCAT1 receptor), xenotropic viruses (excluded from infection of inbred mice [22]; Xpr1 phosphate exporter receptor), and polytropic/mixed polytropic viruses [24], infecting mouse and nonrodent species [22, 23] using Xpr1 as their receptor [25]. Although many endogenous ecotropic and some xenotropic viruses can form infectious particles, the endogenous polytropic MLVs (P-MLVs) do not produce replication competent viruses [22]. However, these sequences are an abundant source for recombination, when challenged with alternative defective or replication competent viruses. The generation of such recombinants frequently results in viruses with improved virulence and the exchange of the viral *env* gene [26–28]. Of significance to this study, C57BL/10 mice express xenotropic MLV from the *Bxv1* locus, which can be a source of viral proteins as well as genetic material [29].

MLV-based vectors were used in initial human gene therapy trials [30] as well as selected CAR-T cell therapies [31]. In multiple clinical trials, including X-linked SCID [32–34], X-linked chronic granulomatous disease [35] and Wiskott-Aldrich Syndrome [36], but not ADA deficiencies [30], insertional mutagenesis resulted in the outgrowth of oligoclonal populations due to trans-activation of proto-oncogenes [37]. Subsequent approaches involving self-inactivating (SIN) vectors [38] or lentiviral vectors have been used to address these outcomes [30]. Alternatively, addressing the integration target-site bias of gammaretroviruses to integrate preferentially at promoter/enhancer regions by altering or eliminating their interaction with host BET protein could alter the oncogenic potential of these vectors [4, 39].

In this study, the genotoxicity of replication-competent M-MLV lacking the IN region required for interaction with the host BET proteins (IN TP^-^) was directly examined using the *MYC/Runx2* transgenic mouse model [20, 21]. Analysis of tumor progression within *MYC/Runx2* mice infected neonatally with MLV that maintain the IN TP^-^ genotype as well as the integration preferences of such mutants in human K562 cells highlights the preferential integration into quiescent states and the strong selective pressure on MLV to maintain the IN tail peptide, through either internal deletions or recombination with endogenous retroviruses. The impact of our findings on the development of MLV-based vectors for human gene therapy is discussed.

## Results

Previously, in vitro studies indicated that the integration bias of MLV IN lacking the tail peptide towards CpG islands and TSS was reduced due to the loss of interaction with host BET proteins [4]. In this study, the effects of virus lacking the BET interaction domain within the IN TP were examined using the mouse *MYC/Runx2* model. This model is advantageous because lymphomas form within 36 days, and lymphomagenesis is accelerated by a further 10 days following insertional mutagenesis by M-MLV at known CIS.

### Generation and characterization of replication competent M-MLV lacking the IN TP

The MLV IN TP region and the *env* coding sequence overlap in alternative open reading frames. In order to analyze the effects of an M-MLV bearing IN C-terminal truncations on the *MYC/Runx2* mouse model, replication competent viruses that terminated the IN protein without altering the expression of the ecotropic M-MLV Env were required.

#### Studies utilizing MLV IN-XN

**Fig 1:**
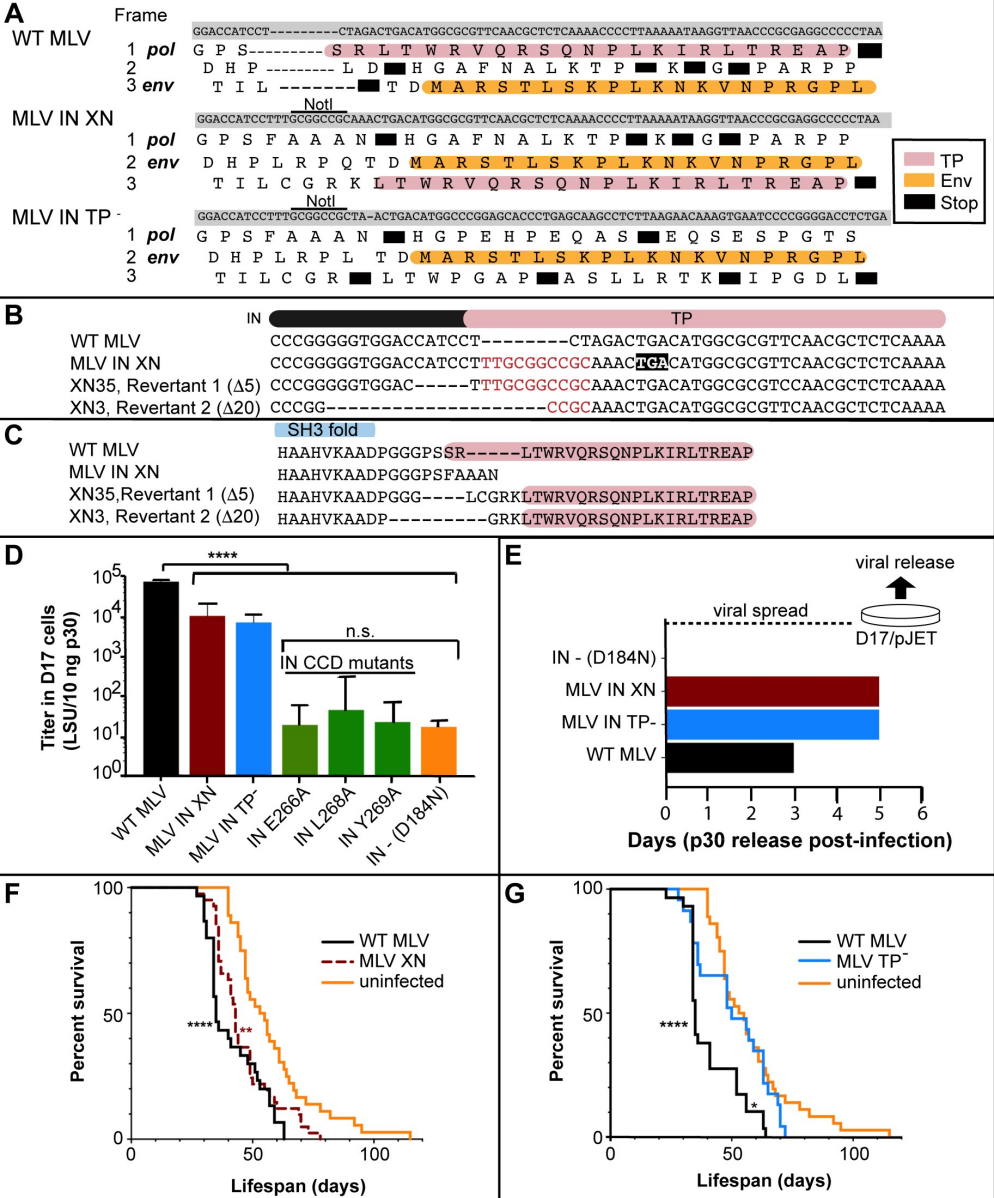
MLV IN TP^-^ constructs and viral characteristics in cell culture and in the *MYC/ Runx2* mouse model. (A) Alignment of the three MLV IN constructs, WT MLV, MLV IN-XN and MLV IN TP- in the overlap region of the IN TP (pink) and Env (orange) reading frames. Black boxes represent stop codons. (B) Viral revertants identified in *MYC/Runx2* tumors infected with MLV IN-XN. The different *pol* sequences are aligned with regions corresponding to the IN (black bar) and TP (pink). NotI linker insertion [40] that generated IN-XN is indicated in red. Deletions (Δ5 and Δ20) with respect to IN-XN are indicated by dash lines. The premature TGA stop codon in IN-XN is shown by black box. (C) Protein alignment of WT MLV, MLV IN-XN and IN revertants. Deletions were localized between the SH3 fold (blue) and the TP (pink) of IN. (D) LacZ titers (LSU, *lacZ* staining units) of the various IN mutant constructs: WT MLV (black), MLV IN-XN (dark red), MLV IN-TP- (blue), IN CCD mutants (green), and IN—D184N (orange). Dunnett’s Multiple comparison test: ****P<0.0001, n.s = no significance. Error bars indicate SEM; n = 3. (E) Viral spread of MLV IN mutants and WT MLV in D17/pJET cells measured by p30 (CA) released into media. Proviral DNA was transiently introduced into cells using DEAE dextran. Viral supernatants were collected at the indicated days and levels of CA were detected by ELISA [86]. (F) Survival curves of *MYC/Runx2* mice infected neonatally with MLV IN-XN. WT MLV (solid black) or MLV IN-XN (dashed brown); the solid orange line is for non-infected control (NC) animals. Log-rank test survival curve comparisons: MLV WT (n = 30) vs. MLV IN-XN (n = 40) P = 0.09; MLV WT vs. uninfected (n = 36), ***P<0.0001; MLV IN-XN vs. uninfected, **P≤0.002 (G) Survival curves of *MYC/Runx2* mice infected neonatally with MLV IN TP^-^. WT MLV (black) or MLV IN TP^-^ (blue); orange is same as in panel F. Log-rank test survival curve comparisons: MLV WT (n = 29) vs. uninfected (n = 36); ****P<0.0001; MLV WT vs. MLV IN TP^-^ (n = 23), *P = 0.006; MLV IN TP^-^ vs. uninfected, P = 0.26.

Initial experiments were performed using the previously reported pNCA-C IN-XN construct [40], which truncated the C-terminal 23 aa of IN while maintaining a viral titer within 2 fold of WT M-MLV in 293T cells [4]. As illustrated in Fig 1A, the IN-XN construct introduces a stop codon within IN, upstream of the *env* coding region, resulting in a frame-shift of the sequence encoding the IN C-terminal region. Survival of *MYC/Runx2* mice infected with WT M-MLV or MLV IN-XN in comparison to an uninfected control was monitored by Date of Death (DoD) over a 115-day period following neonatal injection. The survival curves of WT (n = 30) and IN-XN (n = 40) infected mice were statistically indistinguishable (P = 0.089) using the Log-rank test but both showed significant differences using the same test to the uninfected control (****P<0.0001, **P<0.0021 respectively) (Fig 1F). Median survival times of WT and IN-XN infected mice were 35 and 43 days, respectively, and both succumbed to tumors significantly faster than the uninfected control (median survival 54 days). To investigate further, IN-XN virus isolated from the tumors of three infected mice was introduced into 293mCAT human cells that express the mouse ecotropic receptor [41], which facilitated the isolation of infectious virus in the absence of endogenous mouse viruses. Remarkably, the viruses transferred to 293mCAT cells from two independent mice (XN3; DoD 30d and XN35; DoD 70d) harbored deletions of 20 and 5 bases, respectively (Fig 1B). These deletions counteracted the IN stop codon and restored the C-terminus, encoded in an alternate reading frame, onto the IN protein (Fig 1C). Functionally, such deletions alter the spacing between the IN C-terminal domain (CTD) SH3 fold and the region of the C-terminus that becomes structured upon binding to the host Brd ET domain (Fig 1C) [41]. DNA from the XN2 mouse (DoD = 41d) maintained the IN-XN genotype. From this we noted that reversion to the WT TP sequence did not necessarily correlate with early-onset DoD.

#### Studies utilizing MLV IN TP^-^

In order to address the complication of small deletions restoring the TP to the IN protein, a second construct that codon optimized the *env* reading frame, pNCA-C IN TP^-^, was generated. This construct design eliminated the coding potential of the IN TP within the *env* overlapping region and incorporated multiple stop codons into the non-*env* reading frames, thus eliminating the potential to restore the WT IN sequence through simple deletion (Fig 1A). Single-round infection of D17 cells with MLV IN TP^-^ confirmed that the viral titer was equivalent to MLV IN-XN; in this assay, a 10-fold decrease compared with the wild-type (WT) MLV was observed (Fig 1D). However, highlighting the infectious potential of both MLV IN-XN and TP^-^ viruses, their titers were 3-orders of magnitude greater than the D184N IN mutant virus that carried the Asp>Asn substitution in the enzyme active site [41]. We were also interested in examining the effects of three amino acid substitutions in the IN CCD (E266A, L268A, and Y269A) that were previously reported to disrupt MLV IN interaction with Brd2 proteins using co-immunoprecipitation experiments [42]. However, the viral titers of MLV bearing IN E266A, IN L268A, and IN Y269A were equivalent to the catalytically inactive IN D184N mutant (Fig 1D). Thus, tissue culture and animal studies with these viruses were not possible.

Viral passage of IN-XN and TP^-^ viruses in D17/pJET cells, which express the ecotropic mCAT receptor [43], displayed similar infection time courses, with viral capsid proteins (CA) detected in the medium on day 5. This is a delay of two days compared to the WT MLV (Fig 1E). Competency of the IN TP^-^ virus was further confirmed by western blot analysis of the viral CA, IN, Env proteins released from D17/pJET producer cell line (S1 Fig), with both Env (74 kDa) and CA (30 kDa) detected in the media of cells infected with either MLV IN TP^-^ or WT MLV virus. The truncation of the IN TP was stable after passage in tissue culture (S1 Fig, lane 1), and the predicted decreased molecular weight compared with WT IN was maintained (S1 Fig, lane 2). As expected, no viral proteins (Env, CA or IN) were detected for the replication-defective pNCA-C IN D184N virus (S1 Fig, lane 3).

**Fig 2:**
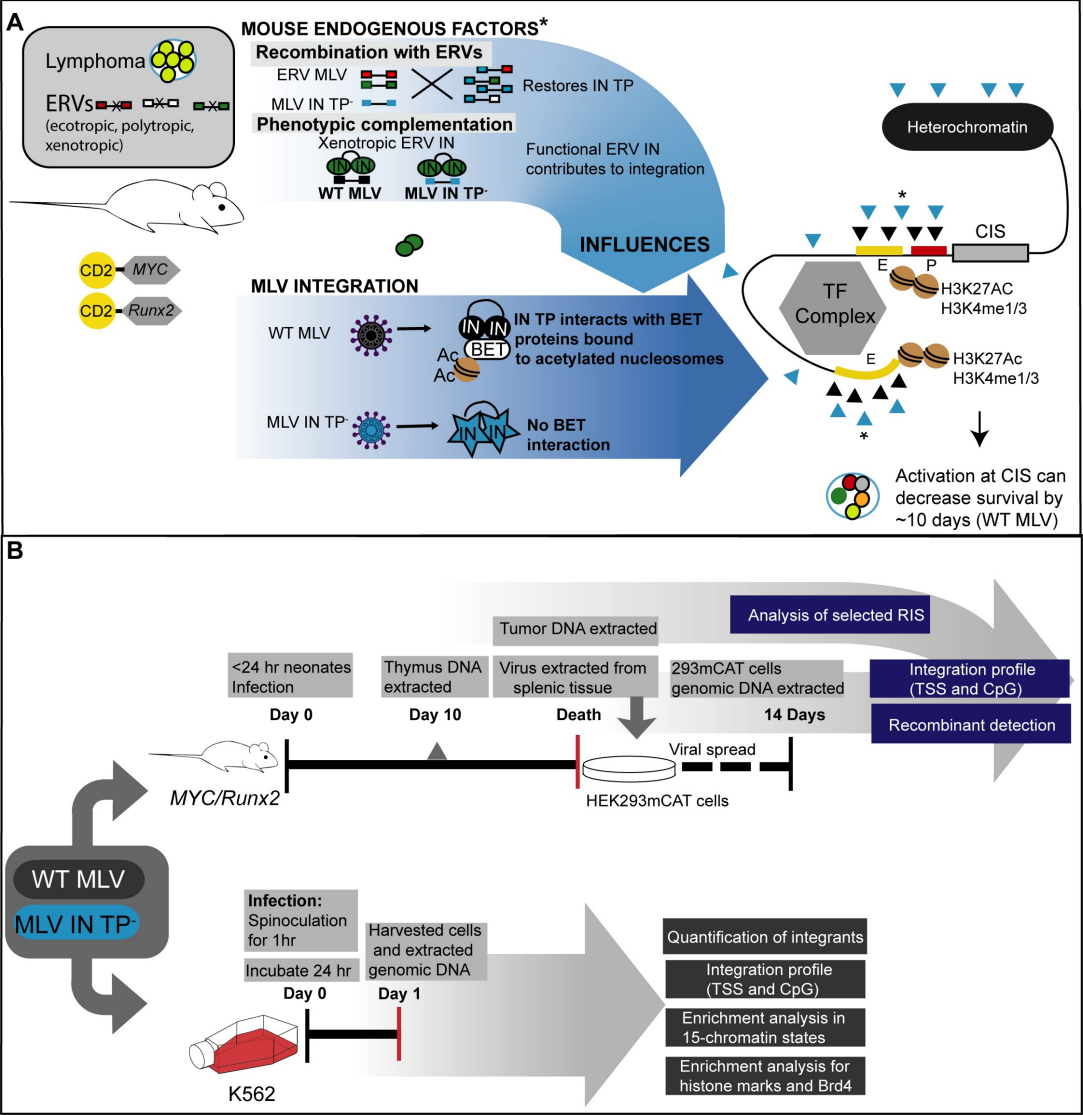
*MYC/Runx2* mouse model and K562 cell study workflows. (A) Overall outline of the *MYC*/Runx2 mouse and MLV infection. Transgenic expression of the *MYC* and *Runx2* genes from the CD2 promoter in the C57BL6 x CBA/Ca mice results in the production of lymphomas. The mice encode endogenous retroviruses (ecotropic, polytropic and xenotropic MLVs) that can influence exogenous infecting viruses through either recombination or protein complementation if functional GagPol proteins are expressed (marked by asterisk). Infection with WT MLV (black circles) results in the IN protein interacting with host BET proteins (white oval) that bind acetylated histone marks. WT IN:BET protein interactions bias integration events (black triangles) towards promoters (P, red rectangle) and enhancers (E, yellow rectangles) through the BET nucleosome mark recognition. Integration events at known common insertions sites (CISs) result in early onset lymphomagenesis. Experiments in this manuscript examine the effects of infection of MLV IN TP^-^ virus that has lost the interaction with the BET proteins (blue stars). Experiments map the positions of IN TP^-^ integrations (blue triangles) with respect to promoters, enhancers, chromatin marks and known CISs in relation to the time of tumor development. (B) Schematic of experiments performed with the *MYC/Runx2* mice (top) and K562 cells (bottom). Details of each experiment are found in Materials and Methods.

Having verified that the MLV IN TP^-^ virus was replication competent and that the truncation was stable, survival experiments were performed in *MYC/Runx2* mice to determine if this optimized construct affected tumorigenesis (Fig 1G and Fig 2). As expected [19–21] (Fig 1F), mice infected with WT MLV exhibited significantly poorer survival than the uninfected controls (Log-rank test: ****P<0.0001; (Fig 1G)), while the lifespan of MLV IN TP^-^ infected mice was extended compared to WT MLV (P = 0.006) (Fig 1G). Interestingly, the survival curve of MLV IN TP^-^ infected mice (n = 23) showed no significant differences (P = 0.26) from that of the uninfected mice (n = 25), in contrast to the observed curve for the mice infected with MLV IN-XN virus (Fig 1F). Of note, the survival curve for IN TP^-^ infected mice showed a biphasic trend with one third of the mice developing tumors comparatively early. Additionally, all of the IN TP^-^ mice were deceased by day 72, while some uninfected mice survived until day 115. The median survival times for untreated mice as well as those infected with WT MLV or MLV IN TP^-^ were 53, 35, and 50 days, respectively. These observed phenotypic differences suggest that infection with MLV IN TP^-^ delayed tumorigenesis compared to WT MLV in this mouse model.

### Analysis of integration site preferences in *MYC/Runx2 mice* infected with WT or TP^-^ MLV

Previous studies showed that truncation of IN TP decreased preferential integration at TSS and CpG islands by >50% in tissue culture [4, 15], and experiments were performed to determine if this was also the case in the *MYC/Runx2* mice. Fig 2 outlines the overall *MYC/Runx2* mouse model (Fig 2A) and workflow for analysis of MLV integration sites (Fig 2B), which were bioinformatically mapped from genomic DNA samples from *MYC/Runx2* mouse tumors following ligation-mediated PCR (LM-PCR) and next generation sequencing (NGS) (Table 1). Initially, libraries were generated from tumors from 4 representative mice infected with WT MLV (WT6, 8, 10, and 12), 5 representative mice infected with MLV IN TP^-^ (TP^-^4, 6, 7, 9, and 16), as well as one non-infected control (NC) mouse (Table 1). Integration sites were initially mapped to +/- 1 kb of TSSs and CpG islands, as described previously [44]. TSS and CpG island-proximal integration frequencies were 11.8–13.4% and 11.9–14.3%, respectively, for WT tumors. Surprisingly, tumors from 4 of the 5 *MYC/Runx2* mice infected with MLV IN TP^-^ (TP^-^4, 6, 7, and 9) showed similar preferential integration at TSSs and CpG islands to WT MLV (10.2–11.9% and 11.3–13.4% respectively). In contrast, MLV integrations from TP^-^16 tumor at TSSs and CpG islands were markedly lower compared to other tumors from IN TP^-^ and WT MLV-infected mice. MLV integration sites proximal to TSSs and CpG islands from all tumor samples were statistically different compared to the NC control (Table in S1 Table). MLV integrations from TP^-^16 were statistically different from WT6, even though each animal had the same DoD (Day 34, Table in S2 Table) (Fisher’s test, P< 0.001, Table in S1 Table). Furthermore, the percentage of MLV TP^-^16 integrations at TSSs (7.3%) and CpG islands (6.3%) was respectively 1.7- and 2.0-fold lower than the mean from all WT MLV tumors at these markers (TSSs = 12.4% ± 0.35 and CpG = 12.9% ± 0.57). While the integration profiles of MLV TP^-^16 are consistent with the loss of the IN TP region, insertions from tumors TP^-^4, 6, 7, and 9 were more similar to the WT virus. Unlike the IN-XN construct, the IN TP construct cannot revert to functional IN through deletions in the viral genome; however, recombination with endogenous retroviruses could have restored the IN TP in TP^-^4, 6, 7, and 9 tumors.

**Table 1:** MLV integration site mapping of WT MLV and MLV IN TP^-^ tumors from *MYC/Runx2* mice.

Tumor	Unique sites	TSS +/- 1kb (%)	CpG +/- 1kb (%)
WT6	17527	2352 (13.4)	2369 (13.5)
WT8	4605	549 (11.9)	547 (11.9)
WT10	5721	678 (11.8)	697 (12.2)
WT12	1474	181 (12.3)	211 (14.3)
TP^-^4	3565	427 (11.9)	477 (13.4)
TP^-^6	2402	274 (11.4)	282 (11.7)
TP^-^7	8139	912 (11.2)	986 (12.1)
TP^-^9	1641	169 (10.2)	186 (11.3)
TP^-^16	536	39 (7.3)	34 (6.3)
NC	2095	50 (2.4%)	56 (2.7%)

### Detection of M-MLV recombination with ERVs

To verify the structure of the virus that persisted in TP^-^16 tumors and to investigate the cause of WT-like behavior of TP^-^4, 6, 7, and 9 tumors, the samples were analyzed for recombination with ERVs (Fig 2A). Recombinants were detected using PCR with primer pairs that included a primer for each of two different classes of known ERVs (polytropic and xenotropic) and an M-MLV primer (S2 Fig, Table in S3 Table, [24]). Amphotropic Env primers were included in the analysis as a control for amplification of laboratory constructs [45].

#### Analysis of recombinant MLV through infection of 293mCAT cells

**Fig 3:**
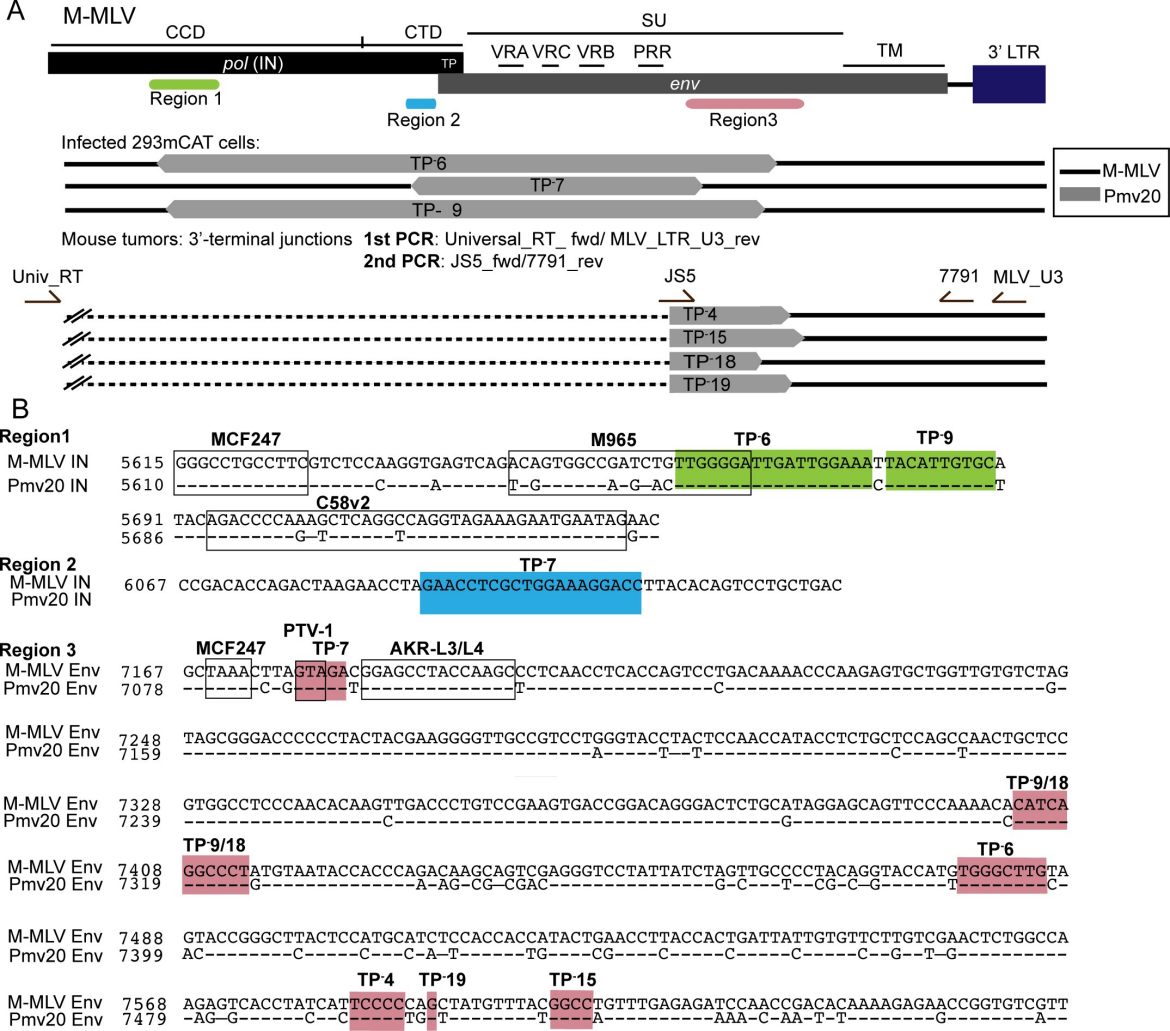
Recombinants in MLV IN TP^-^16 tumors and 293mCAT cells infected with tumor derived viruses. (A) Diagram of the breakpoints of the recombinants in *pol* and *env* regions. The three regions where breakpoints were localized are indicated; region 1 (green), region 2 (blue) and region 3 (salmon). Recombinants isolated from mouse tumors and infected 293mCAT cells are grouped as indicated. Segments with homology to *Pmv20* are indicated in grey and M-MLV is indicated in black. For infected 293mCAT cells, primers used to identify the recombinants are in S2 Fig. For recombinants identified in tumors, the nested PCR primers used to identify the 3’ breakpoints are shown in the diagram. The dashed black line indicates undetermined 5’ junction point for those revertants. (B) The breakpoints of recombinants on the alignment of the M-MLV and *Pmv20* are shown and arranged by region. Previously reported recombinants [46] are indicated in black boxes in region 1 and region 3 (PTV-1). Coloring of the three regions are as indicated in panel 3A. Crossover regions within individual tumors are labeled.

To facilitate this analysis, infectious virus isolated from tumor cells was used to infect human 293mCAT cells that express the mouse ecotropic virus receptor. Env recombinants with polytropic and xenotropic MLV would also be infectious on this cell line. Transferring the virus to 293mCAT cells eliminates the potential for background amplification products from ERVs in mouse cells. PCR analyses performed on viruses derived from TP^-^4, 6, 7, 9, 16 tumors using primers specified in Table in S3 Table are shown in Fig 3 (corresponding DNA gels in S3 Fig). Viral PCR products were detected for TP^-^6, 7, and 9 using the RT_universal_fwd primer and two independent reverse polytropic specific primers (Polytropic_JS4_rev and Polytropic_JS5_rev) (S3A and S3B Fig). The absence of mouse contamination in the 293mCAT DNA was confirmed with negative PCR results for intracisternal particle A (IAP) and mouse mitochondrial cyclooxygenase-2 (mCOX2) DNA sequences (S3C and S3D Fig). Only TP^-^9 yielded a PCR product using the RT_universal_fwd primer paired with either the Amphotropic_rev or the Xenotropic_JS10_rev, but the quality and quantity of this product was insufficient for subsequent analysis.

For these studies, it is of interest to define the crossover junctions within recombinants, as these directly address the potential restoration of the IN TP and receptor recognition of the subsequent virus. Thus, 3’ and 5’ sequence junctions of M-MLV/polytopic ERV recombinants were determined. Polytropic ERV (P-ERV) DNA segments from TP^-^6, 7, 9 had close homology to the P-ERV *Pmv20* [46, 47]. The 5’ junction points for the recombinants were found to be within the IN region, with those of TP^-^6 and TP^-^9 within the IN CCD (Fig 3A and 3B, region 1, green) and that of TP-7 within the IN CTD (Fig 3A and 3B, region 2, blue). The 3’ junction points for all corresponded to the C-terminus end of the surface (SU) subunit of Env glycoprotein. In summary, the segments that recombined into the M-MLV TP^-^ virus contain the WT IN TP sequence, resulting in the restoration of expression of full-length IN during viral spread in the mice.

#### Analysis of recombinant MLV in mouse tumors

Having shown that recombination had occurred in some of the mice infected with MLV IN TP, tumors from other mice with early DoD were investigated to see if recombination events had also occurred. Tumor DNA samples from MLV IN TP^-^4, 12, 13, 15, 16, 17, 18, and 19 (Table in S2 Table) were thus analyzed by nested PCR. The primary PCR product was generated using a forward primer that hybridized to all MLV classes (RT_universal_fwd) and a reverse M-MLV primer (MLV_LTR_U3_rev) that amplifies 3’ M-MLV DNA sequences including IN and Env. For the second round, polytropic recombinants were detected using a Polytropic_JS5_fwd primer and an M-MLV reverse primer (7791_rev) (Fig 3A, bottom). Recombinant viruses were detected in tumor samples TP^-^4, 15, 18 and 19, and were successfully sequenced to identify the 3’ junction points (Fig 3B). However, the 5’ junction breakpoints were not identified in these recombinants. As above, the polytropic segment of these recombinants was homologous to *Pmv20*. The 3’ breakpoints for the TP^-^ recombinants varied throughout the C-terminus of the Env SU (Fig 3B (region 3; bottom)). The 3’ breakpoints of TP^-^9 and TP^-^18 could not be distinguished in this analysis.

The recombinants identified within the IN (Region 1) had crossover junctions distinct from that of the MCF247 and C58v2 recombinant MLVs, with TP^-^6 having partial overlap with the previously identified crossover region of M965 MLV (Fig 3B, black boxes) [46]. Similarly, within Region 3, the crossover identified in TP^-^7 partially overlapped that previously described for PTV-1 [46]. Notably, the presence of recombination did not correlate with tumorigenesis in the *MYC/Runx2* mouse. For example, TP^-^16 with a DoD of 34 days had no detectable recombinants in any of its DNA samples, while TP^-^4, 6 and 9 all had recombinant virus and DoDs of 50, 57 and 63 days, respectively (Table in S2 Table).

### The integration profile of MLV IN TP^-^16 tumor is distinct from that of WT MLV

**Fig 4:**
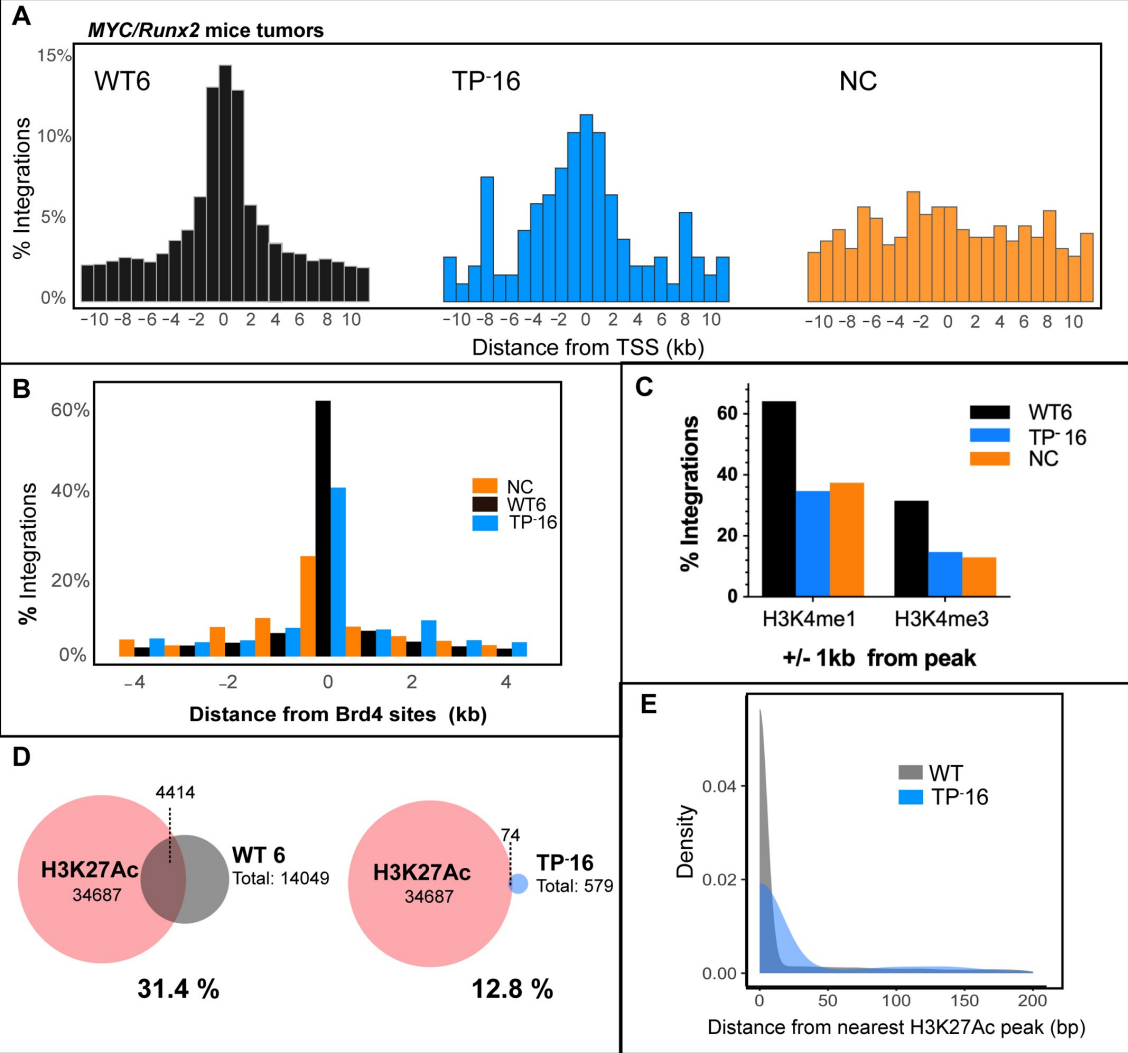
Comparison of MLV integration profiles of tumors from IN TP^-^16 and WT6 mice. In all panels, the IN TP^-^16 integrants are indicated in blue, WT6 in black/grey, and non-infected control (NC) in orange. (A) Histograms of MLV integration profile with respect to TSSs. (B) Association of RIS with Brd4 binding regions (annotated from GSM1262345). (C) The percentage of RISs that overlap within +/- 1 kB from peaks of histone marks H3K4me1 and H3K4me3. (D) Venn diagram of overlap of H3K27Ac peaks (ENCFF974HMO) with RISs. The dash lines indicate the number of RISs overlapping with H3K27Ac. (E) Density plot of RISs from nearest H3K27Ac peaks. The average WT RISs from all tumor samples (grey) is plotted against TP^-^16 (blue).

Analysis of the MLV IN TP^-^ genome within TP^-^16 tumor DNA did not detect recombination with ERVs and indicated decreased viral integrations at TSSs and CpG islands (Table 1). We therefore sought to determine detailed profiles of WT and TP^-^ MLV integration in tumors that arose with similar kinetics, as evidenced by the same 34 day DoD of WT6 and TP^-^16 animals (Table in S2 Table; Fig 4). As expected, WT MLV integrated symmetrically around TSSs (black, Fig 4A left) compared to the NC sample (orange, Fig 4A right), with the majority of the integrants located within 1 kb of TSSs. TP^-^16 distribution of integrants around TSSs was asymmetric and more dispersed compared to WT6 (Fig 4A blue, center), with comparatively increased integration events located ±8 kb from TSSs. Comparison of the MLV integration profile in these tumors with Brd4 binding sites (data taken from ENCODE ID GSM1262345, murine AML MLL-AF9/NrasG12D cells) indicates that MLV IN TP^-^16 integrations at Brd4 sites was decreased by approximately 20% compared to MLV WT6 (Fig 4B). Additionally, approximately 50% fewer TP^-^16 integrations were observed +/- 1 kb from the histone modifications H3K4me1 and H3K4me3 associated with active chromatin when compared to WT6 (ENCODE IDs ENCSR000CCI and ENCSR000CCJ, respectively), and were at a levels that were comparable with the NC (Fig 4C). Statistical analysis of the WT and TP^-^16 integrants with respect to Brd4, H3K4me1 and H3K4me 3 is presented in Table in S1 Table. H3K4me1 and H3K4me3 are considered MLV supermarkers [48], with H3K4me3 associated with nucleosome-bound BET protein [49].

In addition to the histone marks studied above, BET proteins associate significantly with active enhancer features, specifically acetylated histone tails. In the human lymphoma cell line Ly1 DLBCL, 79.1% of H3K27Ac sites overlap with Brd4 and 92.2% of chromatin bound Brd4 is at regions of the H3K27Ac active enhancer mark [50]. MLV integrations are also reported to be highly enriched at H3K27Ac sites [5, 12, 42, 51]. 31.4% of MLV integration sites from WT6 tumors were within H3K27Ac sites (ENCODE ID ENCFF001KYG) (Fig 4D), whereas only 12.8% of the MLV IN TP^-^16 integration sites overlapped with H3K27Ac sites. Furthermore, all of the WT samples analyzed had similar overlap with H3K27Ac sites (31.2% for WT8, 35.3% for WT10, 37.1% for WT12) (Table in S4 Table). The distribution of the distance between retroviral integration sites (RISs) and the nearest H3K27Ac peak was significantly broader for TP^-^16 than for the cumulative RISs from all WT tumors (Wilcoxon Rank Sum, p<2.2 x 10^−16^; Fig 4E).

Viruses extracted from MLV IN TP^-^16 tumor were used to infect 293mCAT cells and integration site analysis was performed after 14 days. The integration profile in 293mCAT for MLV IN TP^-^16 paralleled that of MLV IN-XN (Table 2). Overall, these integration profiles indicate that the viral population from the TP^-^16 tumor maintained the MLV IN TP^-^ genotype within the mouse and that the integrants are distributed further away from promoter and active enhancers compared to WT MLV.

**Table 2:** Comparison of integration sites in 293mCAT cells of MLV IN XN and MLV IN TP^-^ derived from TP^-^16 tumor.

Sample	Unique sites	TSS+/-1kb (%)	CpG+/- 1kb (%)
WT^a^	64828	14208 (21.9)	18864 (29.1)
IN XN^a^	37638	2029 (5.4)	3171 (8.4)
IN TP^-^16^b^	47093	2959 (6.3)	4541 (9.6)
RIC^c^	10000	169 (1.7)	270 (2.7)

^a^ Viral spread through plasmid transfection of 293mCAT cells

^b^ Viral spread by infection of viruses extracted from mouse tumor

^c^RIC, random integration control

### Integration sites within the MLV IN TP^-^16 tumors localize to known MLV integration common insertion sites (CISs)

**Fig 5:**
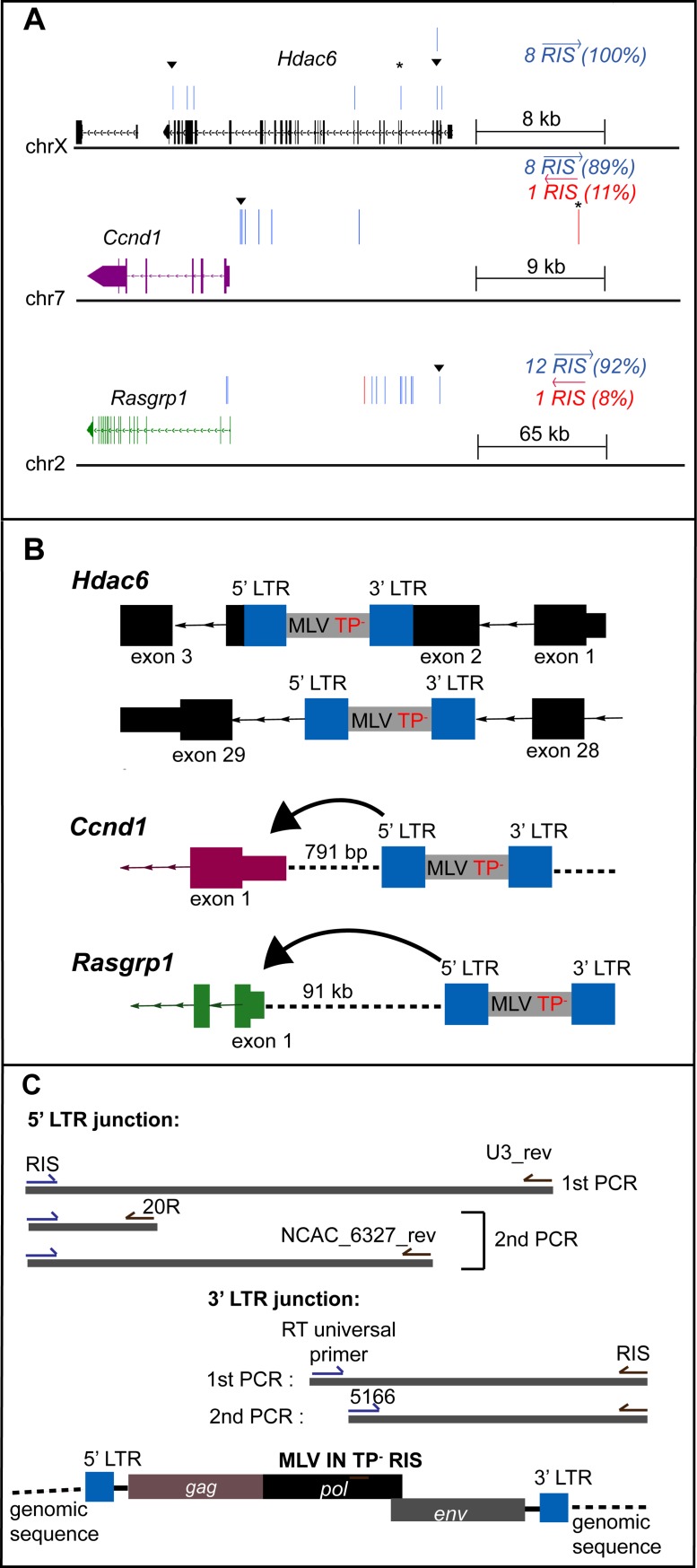
Analysis of the top targeted integration sites within the IN TP^-^16 tumor. (A) Orientation bias of RIS in three CIS genes: *Hdac6* (black), *Ccnd1* (purple), *Rasgrp1* (green). The exons and introns are represented in boxes and lines with arrow that show strand orientation respectively. RIS is represented by a vertical bar and differentially colored based on orientation (blue forward, red reverse) relative to the plus-strand DNA. Gene structures are derived from Integrated Genome Browser (mm10). (▼) denotes the top copy number RIS analyzed. (B) Diagram of the TP^-^16 RISs relative to the closest CIS gene. The integrated MLV is depicted in grey, with LTR indicated in blue. Coding regions of CIS are represented as in panel A. Intergenic regions are represented in dashed lines. Direction and distance between 5’ LTR and TSS are indicated. IN regions verified to maintain the TP^-^ phenotype are indicated. (C) Schematic diagram of the nested PCR utilized to isolate the RIS from mouse tumor DNA. Primers used in the first and second round PCRs for the 5’ and 3' LTR junction points of RISs are included in the diagram. Sequences of all oligonucleotides are described in Table in S2 Table.

Integration site copy number is representative of predominant integrants that clonally expand during tumorigenesis [20]. Table in S5 Table summarizes the key MLV integrants present in the IN TP^-^16 tumor. The top 25 CISs for WT MLV in the *MYC/Runx2* mouse have previously been identified [20]. Within the IN TP^-^16 tumor, integrants localized at 11 of the 25 CIS (S5 Table). Significantly, of the top ten copy number integrants, seven were within these previously identified loci (Table in S5 Table). The viral integrants from the TP^-^16 tumors at three of these CIS genes, *Mapk13*, *Ccnd1*, *Hdac6*, is shown schematically in Fig 5A & 5B. Insertions at all three genes showed an orientation bias for the genomic (+) strand (mm10). For *Ccnd1* and *Rasgrp1*, this orientation bias is antisense to the host gene transcription, which is consistent with enhancer insertional activation reported previously [52–54]. Interestingly, the *Rasgrp1* cluster of integrations mapped >91 kb upstream of the *Rasgrp1* promoter, whereas for *Ccnd1*, the high copy number insert was located 791 bp from the promoter (black triangle, Fig 5A). It is striking that three independent insertions in *Hdac6* were highly abundant, and all were within the gene: the first disrupted exon 3 and corresponded to the N-terminus of the protein, while two abundant insertions localized within introns spanning the gene (introns 5 and 28) (Table in S5 Table).

#### Integrants at CISs encode the IN TP^-^ genotype

It was of considerable interest to verify that the MLV inserted into these three genes maintained the IN TP^-^ genotype. Based on the known insertion sites, a nested PCR was developed to amplify the IN region of *pol* with specific host sequences (Fig 5C and Table in S5 Table). Sequencing of the resulting PCR products verified that all four insertions at *Hdac6* (2 insertions), *Ccnd1*, and *Rasgrp1* maintained the parental IN TP^-^ mutation.

### Mapping integrations of MLV IN TP^-^ in K562 cells for 15 chromatin states and histone modifications

**Fig 6:**
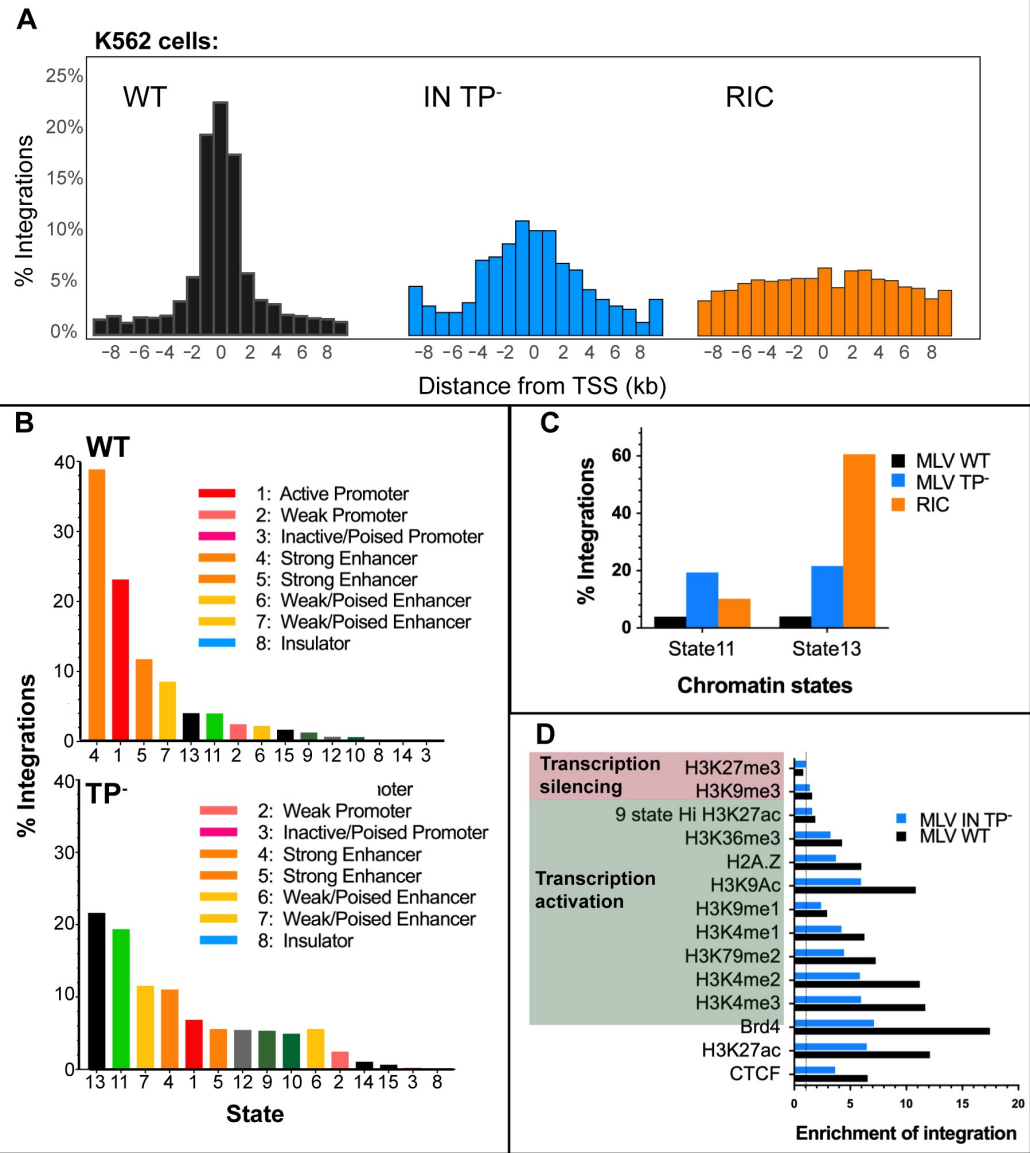
Analysis of IN TP^-^ and WT MLV integration sites in K562 cells. (A) Histograms of MLV integration profile with respect to TSSs. (B) Percentage of RISs in 15-chromatin states [55] in K562 cells: WT (top panel) and IN TP^-^ (bottom panel). Each chromatin state is labeled with corresponding color as indicated. (C) Percentage of RISs in state 11 and state 13 compared to RIC. State 11 is described as weakly transcribed regions and state 13 are heterochromatin regions [55]. (D) Enrichment of integrations in ChipSeq peaks of different histone marks and Brd4 binding regions in K562 cells. Value of enrichment is calculated by dividing the number of RISs with RIC values at each histone mark. The dotted line is the level of enrichment expected by chance. Transcription silencing histone marks are in pink and transcription activating marks are in green.

To further investigate the pattern of MLV IN TP^-^ integration, a single round of infection of leukemia cell line K562 with this virus was performed. As expected, these cells recapitulated the decreased integration percentages at TSSs and CpG islands that was previously observed in 293mCAT cells for the IN-XN construct (Tables 2 and 3, Fig 6A). As previously reported [13], the use of chromatin states provides a different approach to understand the genome landscape. In this approach, clusters of chromatin marks are used to define functional active states of chromosomes, which are specific for each cell line. For K562, 15 chromosome states have been utilized to analyze MLV integration [13], and 85% of the integrants were shown to map to strong enhancer and active promoter regions. The overlap between MLV WT and TP^-^ integration sites and the components of the 15-chromatin state model was investigated in K562 cells. As shown in Fig 6B, 73.8% of MLV WT integrations mapped to the same three highest states that were identified previously [13], which were annotated as active promoter (state 1) and strong enhancer (states 4 and 5). In contrast, MLV IN TP^-^ integrations displayed a divergent preference, with the top two chromatin states being heterochromatin (state 13, (21.6%)) and weakly transcribed region (state 11, 19.4%) (Fig 6B, bottom). Loss of the IN TP decreased, but did not eliminate, integrations at active promoters (state 1) and enhancers (strong enhancers, states 4 and 5, and weak enhancers, state 7), which cumulatively accounted for 35% of the IN TP^-^ integrants and are drastically reduced compared to WT MLV (82%).

**Table 3:** Integration site mapping of single round infected WT and IN TP^-^ MLV in K562 cells.

Sample	Unique sites	TSS+/-1kb (%)	CpG+/- 1kb (%)
WT	4384	1160 (26.5)	1417 (32.3)
IN TP^-^	934	68 (7.3)	98 (10.5)
RIC	10000	169 (1.7)	270 (2.7)

The median chromosomal coverage in K562 for chromatin states 11 and 13 were reported to be 11.3 and 71.4% [55], respectively, which correlates with the observed integrations from the coverage of the computer-generated random integration control (RIC) within these states (Fig 6C). For state 11, corresponding to weakly transcribed regions, loss of the IN TP increased integration frequency 4.8-fold compared to WT (Fig 6C; 19.4% for IN TP^-^; 4.01% for WT MLV). Significantly, this frequency is 2-fold above the RIC, indicating a bias for integration into these weakly transcribed regions (Table S1 Table). Similarly, integration into heterochromatin (state 13), increased ~ 5-fold in MLV IN TP^-^ (21.63%) compared to WT MLV (4.06%). Thus, in the absence of the IN TP, MLV integration preference for heterochromatin and weakly transcribed regions increases, while integration at active promoters and enhancers was diminished.

#### Decreased integration at BET-associated epigenetic marks for IN TP^-^ MLV in K562 cells

Chromosomal states are defined in part through profiling combinations of known histone modifications and the occupancy of various cis-regulatory elements by known protein factors [55]. Directed by the bromodomains, acetylated histone modifications are important in determining BET proteins interactions, however additional marks including H3K4me2/3 are elevated in Brd-bound nucleosomes [49]. MLV integrations strongly associate with H3K4me1, H3K4me2, H3K4me3, H3K27ac, H2Az and H3K9ac modified chromatin [5, 12, 13, 15, 44, 51]. Fig 6D and S4 Fig show the proportion of WT and IN TP^-^ integrants found in regions marked by the various epigenetic marks and proteins associated with integration site preference in K562 cells. ChIP-seq data for all chromatin modifications was obtained from the ENCODE consortium, and enrichment of viral integration over RIC was computed for each modification. Significantly, the largest decrease in enrichment upon loss of the IN TP was associated with Brd4 binding sites (17.5-fold for WT vs 7.1-fold for MLV IN TP^-^) (Fig 6D and S4 Fig). The WT virus was most highly enriched at the epigenetic modifications H3K4me3, H3K4me2, H3K27ac and H3K9ac, which is consistent with previous reports [13]. Interestingly, loss of IN TP resulted in a >45% decrease in fold enrichment at H3K4me3, H3K4me2, and H3K27ac sites, which is consistent with the loss of association with BET proteins.

## Discussion

### *MYC/Runx2* transgenic mouse model and MLV-induced tumorigenesis

The discovery that the MLV IN protein interacts with host BET proteins, directing integration into sites of active expression marked by acetylated chromatin, gave rise to the question of whether the loss of the MLV BET binding site would affect the pathogenesis of the virus. In this study, we used *MYC/Runx2* mice, a transgenic model displaying rapid tumor formation that is further accelerated by MLV infection [18, 19, 21]. These mice were infected with MLV lacking the IN tail peptide. Globally, the infection time course for the IN TP^-^ series showed a biphasic DoD curve, with one-third of the mice developing tumors early, as with WT infection, and two-thirds of the mice developing tumors later, paralleling the uninfected *MYC/Runx2* control mice. We extensively characterized IN TP^-^16 samples, which displayed early tumorigenesis and showed no detectable signs of recombination with endogenous viruses. Characterization of TP^-^16 tumor viral integration sites indicated a profile dispersed away from TSSs and decreased association with H3K27ac marks (Fig 4A and 4C). The IN mutation was preserved within the tumor, and the characteristic target-site profile for IN lacking the TP was maintained following 293mCAT cell infection with IN TP^-^16 tumor-derived virus. However, integrations into CISs known to accelerate tumor formation were also present, suggesting that stochastic integration into an open chromatin hotspot can still provide the positive selection required for tumor outgrowth. It is noteworthy that 7/10 of the highest MLV TP^-^16 copy number integration sites were within known CISs (Table in S5 Table).

### Selective pressure to maintain the IN tail peptide in mice

Our results highlight the strong pressure within the *in vivo* model to maintain the presence of the IN-TP. Interestingly, the TP region overlaps with the Env signal peptide, yet the codon bias within 4070A MLV is maintained towards the IN reading frame [56]. Sequence conservation between gammaretroviruses identified the conserved sequence W(X_7_)PLK(I/L)R within the TP [4]. From our studies, the initial viruses (MLV IN-XN) emerging from the tumors indicated that selective pressures restored the TP through deletion mutations within the virus coding region, removing a small putative linker region within the IN C-terminus to restore the TP in an alternative reading frame. When this option was removed, through optimizing codon usage for the IN/Env region towards the *env* sequence, thereby destroying the coding potential of the TP, circulating virus restored the TP through recombination with endogenous polytropic viruses, with cross-over junctions within the IN and Env coding regions.

### Identification of a linker region between the IN CTD SH3 and TP

The deletion mutations that restored the BET interacting domain onto the IN-XN construct identify a linker region between the IN CTD SH3 fold (ending at KAADPG [3, 40, 57] and the IN BET interaction domain (initiating at IN W390 [4, 15]). After passage in mice, the spacing between the IN SH3 and TP domains is reduced from nine amino acids (GGGPSSRLT) to 3 amino acids (GRK), two of which are not normally encoded by the IN protein. This indicates that the length of this linker region, as well as the composition of the amino acids between the IN SH3 fold and the BET binding site can be substantially altered while still maintaining virus viability *in vivo* [57].

### Recombination with ERVs and temporal effects of tumorigenesis

Recombination with the endogenous polytropic *Pmv20* virus was detected in multiple tumors (IN TP^-^4, 6, 7, 9, 15, 18, and 19). While this recombination restored the IN TP, the presence of recombinant virus did not correlate with mouse DoDs; IN TP^-^16 did not recombine with polytropic virus (DoD 34 days), whereas IN TP^-^4, 6, 7, 9, 15, 18, and 19, which did undergo recombination, had DoDs of 50, 57, 37, 63, 33 and 36 days, respectively. Our analysis cannot determine the point in the infection time course at which recombination occurred, and it is possible that the recombination event for IN TP^-^9, for example, which had a DoD of 63 days, occurred late in tumor development and thus did not have a marked effect on tumor progression. It is interesting to note that following infection with IN TP^-^, no mice survived beyond day 70, whereas some NC mice survived beyond 110 days, suggesting that the presence of a recombinant virus may have affected long-term survival.

### Sequence analysis of viral/host DNA junctions

Interestingly, when the 3’ LTR junctions of 3 of the top 10 RISs (*Mapk13*, *Ccnd1*, and *Hdac6)* were sequenced as a PCR population, heterogeneity was observed at positions +1/+2 downstream of the MLV LTR TCTTTCA terminus. Viral integration involves the cleavage of the LTR terminal dinucleotides, exposing the conserved 3’ CA for strand transfer and generating a 5’ ss AA tail at the 5’ ends of the viral DNA substrate. The observed sequence heterogeneity corresponded with either the predicted host DNA sequence or nucleotides from repair of the 5’ ss viral DNA tail. Heterogeneity at the 5’ LTR-host junction was not observed within the Illumina sequence reads at these genes nor in the 5’LTR analysis of the *Hdac6* gene. The mechanism for this heterogeneity requires further analysis and could reflect simple sequencing compression or a unique repair mechanisms at the viral/host DNA junction in the *MYC/Runx2* mice model, in which p53 activity is reported to be suppressed [18, 19].

### Mechanisms of retroviral enhancer activation

For retroviruses, enhancer activation usually occurs upstream of the gene in the antisense orientation, or downstream in the sense orientation [54]. Indeed, this was the orientation bias observed for integrations into both *Ccnd1* and *Rasgrp1* in TP^-^16 tumor DNA. For *Ccnd1*, the top integrant was in the integration cluster near the promoter (Fig 5A). Similarly, previous studies of WT MLV in *MYC/Runx2* tumors indicated a cluster of insertions at the 5’ end of the *Ccnd1* gene, predominantly upstream of the coding sequence [20]. The proximal cluster initiates overexpression via retroviral enhancer elements [58]. *Ccnd1* has an important role in cell cycle regulation, and overexpression induces the formation of different cancer types [59–61]. For *Rasgrp1*, the integrant analyzed was 91,758 bases upstream of the *Rasgrp1* promoter, and oriented with the viral promoter in the opposite orientation. Activation of *Rasgrp1* would most likely occur through an enhancer activation event and sequence analysis indicated this integrant maintained the IN TP^-^ sequence. It was initially surprising to see the RIS orientation bias >91 kb upstream of *Rasgrp1* promoter. For human T cell leukemia virus-1 (HTLV-1), long-range interactions between target gene promoters and viral enhancers are facilitated through chromatin looping utilizing the host zinc finger binding protein CTCF [62, 63]. The HTLV-1 provirus contains a CTCF nucleotide-binding motif that has been shown to mediate clone-specific deregulation of host transcription from distances up to 300 kb [62], and CTCF-mediated *cis* contacts within the host genome can be as far as 1.4 Mb [62, 63]. Although M-MLV does not encode a known CTCF binding motif, CTCF binding sites have been identified at the promoter region of *Rasgrp1* and ~10 kb downstream from the integration site (ENCODE reference ENCFF310MUQ). CTCF-mediated transcription varies depending on the cell type. Validation of CTCF binding would require circular chromosome conformation capture (3C or ChIA-PET) analysis from the tumors, which is not available for this study. However, the presence of these CTCF binding sites provides a potential mechanism for the MLV enhancer to interact with *Rasgrp1* promoters that are distant from each other, thereby driving overexpression of *Rasgrp1* concomitant with tumorigenesis in these mice [64–66]. For *Ccnd1*, CTCF-dependent long-range loops have been identified that reposition distal clusters of retroviral insertions, driving gene activation [58].

### Biased integration orientation within the HDAC6 gene

The *Hdac6* gene also displayed biased integration in an orientation opposite to that of transcription, however these integrants were within the *Hdac6* gene. For IN TP^-^16, the three most abundant integrants in the library mapped within exon 3 proximal to the gene 5’ end and within introns at the center and toward the 3’ end of the gene. The results imply a loss of function through oncogenic selection, however the mechanism cannot be determined. *Hdac6* is reported to interact with *Runx2* [67, 68] as well as being involved in multiple cellular processes, including organization of the immune synapse, cell migration, protein degradation, and viral infections [69].

### IN domains involved in BET protein recognition

The absence of the IN TP reduced the integration bias for strong enhancers and active promoters and increased preferences for heterochromatin and weakly transcribed regions. The secondary preference towards active regulatory elements (state 1, 4 and 5) was maintained, which corresponds to median genome coverage of only 2.5%. The observed integration of MLV TP^-^ at active promoter/enhancers could be the result of additional IN sequences interacting with BET proteins [42]. Although our studies indicated that the interaction with BET domains occurs predominantly through the IN TP, others have mapped three amino acid changes within the IN CCD, specifically E266A, L268A, and Y269A, which resulted in the loss of Brd2 interaction as assessed by coimmunoprecipitation [42]. We have analyzed these mutants in the context of single-round infection, which indicated that all three mutant viruses had negligible titers, akin to the IN catalytic mutant virus D184N (Fig 1D). Molecular modeling indicates that these positions are located in the predicted MLV CCD α6 helix (residues 264–270) and are close to the CCD dimer interface (S5 Fig). The model shows the close proximity of residues Y269 and L268 between monomers and thus alanine substitutions of L268 and Y269 could disrupt the multimerization of the CCD. Thus the effects of L268A and Y269A changes on IN binding to Brd2 were plausibly indirect.

### Chromatin marks associated with MLV integration and transcriptionally quiescent regions

Heterochromatin and weakly transcribed regions in the 15-state model share the absence of chromatin marks H3K4me1/2/3, H3K27me3, H3K27ac, H3K9ac and CTCF, with the weakly transcribed state 11 containing low levels of H3K36me3 and H4K20me1 [55]. A more defined model with 25 and 50 chromatin states has recently been described [70], which makes further subdivisions based on an expanded set of chromatin marks. In these, the heterochromatin state is distinguished from the quiescent state by the presence of the H3K9me3 mark. While K562 ENCODE data is not available for the full expanded set of chromatin marks, we observed no significant fold enrichment of WT MLV or IN TP^-^ integrations at H3K9me3. Therefore, most of the MLV IN TP^-^ integrations in heterochromatin would reasonably be categorized as targeting the quiescent chromatin state. Similar to the heterochromatin state in 15-chromatin scale, the quiescent state is defined by the large absence of any histone modifications and is transcriptionally inactive with low annotated non-coding and coding transcripts [70, 71].

### Recognition nucleosomes by MLV IN

This is the first study to define where MLV integration is directed in the absence of the BET protein interaction, beyond noting a decreased bias away from active promoters and enhancers [12, 13, 72]. Two models could explain the integration preference into regions with limited modified histones observed for State 11. In the first model, MLV IN may display an innate recognition of unmodified histone tails. In the second model, modified histones may present a steric hindrance for IN binding. Both MLV and prototype foamy virus (PFV) encode an N-terminal extension domain (NED) [2, 8] though PFV IN does not encode a homologous TP. Two loop regions within the PFV IN CCD-CCD dimer interface interact with the H2A-H2B heterodimer, specifically with the C-terminal helix of H2B and N-terminal of H2A [73–75]. Binding to the nucleosome results in a 7Å deformation of the target DNA and ultimately drives viral integration into heterochromatin regions, Lamin A/B1 rich-regions, and intergenic regions [73–76]. Transposing the loop regions of the PFV IN CCD that are implicated in the H2B interaction onto MLV IN resulted in viral titers equivalent to those of the catalytically inactive MLV D184N implying that these surface exposed loops in MLV IN are integral to IN stability or sites of secondary interactions. H2A and H2B are the most diverse histones, which along with distinct post-translational patterns, contribute to the complexity and variability of H2A-H2B dimers [77]. It is possible that the MLV IN CCD-CCD dimer may interact with a specific variant of H2A-H2B heterodimer with distinct modifications independent of the BET proteins.

### Potential of phenotypic complementation of IN by ERVs

In the mouse model, we cannot exclude a role for phenotypic complementation of IN function through co-packaging of TP^-^ Gag-Pol with full-length Gag-Pol expressed by ERVs in tumor cells, specifically transcomplementation by endogenous Gag-Pol proteins from either the *Emv2* [22, 78] or the xenotropic MLV *Bxv-1* loci. C57BL/10 mice have been documented to express xenotropic MLV at low levels *in vivo*, which can be induced in tissue culture with IdU [29]. Thus, although the xenotropic Env would exclude infection by these endogenous viruses, complementation of the IN protein *in trans* cannot be eliminated. This could result in the low level of bias towards the TSS integration observed within the *MYC/Runx2* tumors in the absence of IN TP region recombination events. This issue of transcomplementation by xenotropic or ecotropic MLV would not be expected to be problematic were IN TP^-^ virus to be used in non-murine cells, including human K562 cells. However, each species has their own ERVs, which are much less likely to contribute to the target-site selection of MLV-based vectors.

### MLV IN TP- vectors for gene therapy

The overall goal of these experiments was to determine whether MLV IN TP^-^ might represent a safer gene delivery vector as compared to MLV harboring WT IN. Importantly, the uniform acceleration of tumorigenesis by MLV in the *MYC/Runx2* mouse was not observed with IN lacking the BET interaction domain at its C-terminus. However, a major limitation of this mouse system is the strong selective pressure to maintain IN-TP function, either directly by recombination or indirectly via transcomplementation. Alternative animal models or assay systems [79, 80] would be beneficial to assess the full potential of MLV IN TP^-^ as a vector in the absence of endogenous elements influencing integration preferences. Our results indicate that removing the TP was insufficient to redirect all integrations away from active promoters and strong enhancers, or to eliminate the stochastic events that can select for oncogenic activation. Ultimately, a modified vector that combines SIN LTRs to eliminate strong viral enhancers [10, 81] with insertions/replacement of the IN TP^-^ to redirect integration to less active regions [39, 82] could decrease vector genotoxicity and overcome current limitations for clinical applications.

## Materials and methods

### Ethics statement

All animal experiments were performed under the EU directive 2010 and UK Animals (Scientific Procedures) Act 1986. This specific study was undertaken on project license number P3C97B34C (awarded to E Cameron) ethically approved by the University of Glasgow (Establishment license number XC2FD842E) Animal Welfare & Ethics Review Board and the UK Home Office.

### Cell lines

Human embryonic kidney 293T cells, 293mCAT cells (expressing mCAT receptor) [83] and canine osteosarcoma D17/pJET cells (expresses the mCAT-1 receptor) [43] were maintained in Dulbecco's modified Eagle medium (DMEM; Gibco #11965) supplemented with 10% (vol/vol) heat-inactivated fetal bovine serum (Atlanta Biologicals # S1245OH) and 1x antibiotic-antimycotic (100 units/mL of penicillin, 100 μg/mL of streptomycin, and 0.25 μg/mL Amphotericin B) (Gibco #15240). The parental 293, 293T and D17 cells were obtained from ATCC. Human K562 cells were acquired from ATCC (CCL-243) and maintained in Iscove’s modified Dulbecco’s medium (IMDM, # 12440079) supplemented with 10% (vol/vol) heat-inactivated fetal bovine serum (Atlanta Biologicals # S1245OH).

### Plasmid and vector construction

The replication-competent M-MLV proviral construct pNCA-C [84] and pNCA-C IN-XN (previously named *in*6215a [40]), bearing a 23-aa truncation of the IN tail peptide (TP) of the C-terminal domain (CTD) was previously described [40]. To generate a codon-optimized pNCA-C-TP^-^, a 137 bp gene block (IDT) was chemically synthesized and amplified using primers NCACXN_ScaI6330_rev and NCACXN_NotI6220_fwd. Overlapping PCR of this fragment with a ScaI-ClaI fragment from pNCA-C (generated using primers NCAC_8290_rev and NCACXN_6327_fwd) resulted in a NotI-ClaI fragment, which was exchanged into NotI/ClaI digested pNCA-C IN-XN. Generation of the pNCA-C IN D184N was previously described [41]. Sequences of all oligonucleotides are provided in Table in S3 Table.

The three residues implicated for BET protein binding (MLV IN E266, L268, Y269) were substituted to alanine using overlapping PCR with KOD polymerase [42]. PCR of the first fragment was amplified with primer 102510NdeIINteinIN forward and point mutant specific reverse primer (E266A_rev, L268A_rev, Y269A_rev) and the second fragment was amplified using the point mutant specific forward primer (E266A_fwd, L268A_fwd, Y269A_fwd) and the 102510XhoIInteinIN1-407 reverse. The overlapping PCR fragment was introduced into pNCA-C using the HindIII and PmlI sites. All mutations were verified using Sanger DNA sequencing.

### DEAE-dextran transient transfection of proviral DNA clones in D17pJET cells

Transient expression of the pNCA-C based proviral constructs was performed as previously described [3, 85] using 500 ng pNCA-C based plasmids. Tissue culture supernatant was monitored for viral spread using enzyme-linked immunosorbent assay (ELISA) against MLV p30 [86]. Cultures were maintained for at least 14 days prior to analysis.

### LacZ viral titer assay and single round infection in D17 cells

2 x 10^6^ 293T cells were transfected with 0.8 μg pMD2.G (Addgene) expressing the vesicular stomatitis virus glycoprotein (VSV-G), 0.8 μg pRT43.2Tnlsβ-gal [87] a retroviral packaging vector expressing *lacZ* and 0.8 μg M-MLV viral genome with wild-type (pNCA-C) or MLV TP^-^ (pNCA-C TP^-^) using Fugene 6 (Promega #E2691) overnight as directed by the manufacturer [88]. Viral supernatant was collected, filtered through a 0.45 μm syringe and viral particles were quantified using ELISA against the MLV p30 [86]. 1x10^5^ cells D17 cells on 3.5-cm gridded plates were infected with media containing 10 ng of CA in 2 ml DMEM in the presence of 8 μg/ml polybrene. Medium was replaced with fresh DMEM after 24 h of infection. Cells were stained for LacZ expression as previously described [45].

### Western blot

Viruses were collected from D17/pJET viral producer cell lines. For CA and Env western blot, 2 ml of viral supernatant was spun at 15,000 x g for 30 min and the viral pellet was resuspended in 20 μl phosphate buffer saline (PBS). Samples were run on a 10% SDS-PAG and transferred to polyvinyl difluoride (PVDF) membranes using Bio-Rad Trans-Blot Turbo Transfer System. Immunoblots were developed using goat anti-p30 (CA) (1:2000, 81S-263) and goat anti-Env (1:1000, 80S-019) (Quality Biotech) with bovine anti-goat HRP (80S-035-180) as secondary antibody (1:10,000). For IN western blot, 10 ml of viral supernatant was pelleted at 15,000 x g for 30 min and the proteins were visualized using 1:1 mix (1:1000) of antiserum from Rabbit 3 and Rabbit 4, Bleed 5 with goat anti rabbit HRP (Pierce #31460) as secondary antibody (1: 5,000) [89].

### Infection of MLV into *MYC/Runx2* mice

The *MYC/Runx2* transgenic mice are on a C57BL6 x CBA/Ca background. Infection and maintenance of the mice was as described [21]. For WT and mutant MLV, viruses were obtained from 293mCAT cells to avoid recombination with endogenous viruses prior to infection. Briefly, mice were inoculated intraperitoneally with virus isolated from tissue culture supernatant (10^5^ TCID_50_) within 24 h of birth. Date of Death (DoD) was monitored over a 115-day period. For each mouse, a tissue fragment was thawed from liquid N_2_, minced and incubated in medium at 37°C for 2–3 hrs. The medium was spun at 1,200 rpm and then filtered (0.45μm) before adding to 293mCAT cells. Cells were cultured for at least 7 days before harvest for DNA isolation. Amplification of the integrated MLV genomes from the 293mCAT cells was performed using primers 4924 and 7791, previously named 3807 and 6320, respectively [45] (Table in S3 Table). PCR products were cloned using TA cloning. Individual colonies from mice XN-2, 3, and 35 were selected and sequenced for presence of the TP coding region.

### Detection of mouse DNA

Genomic DNA isolates from 293mCAT cells were analyzed for mouse DNA contamination by examining for mouse intracisternal particle A (IAP) and mouse mitochondrial cyclooxygenase-2 (mCOX2). The primers used were mouse_IAP_fwd (ATAATCTGCGCATGAGCCAAGG) and Mouse_IAP_rev (AGGAAGAACACCACAGACCAG) [90], and primers used for COX2 were Mouse_mt__COX2_fwd (5′ TTC TAC CAG CTG TAA TCC TTA 3′) and Mouse_mt_COX2__rev (5′ GTT TTA GGT CGT TTG TTG GGA T 3′) [91]. PCR analysis was performed using KOD HotStart.

### Analysis of M-MLV recombination with endogenous retroviruses

Detection of recombination with ERVs utilized primers recognizing M-MLV, polytropic, xenotropic ERV and the RT_universal_primer (Table in S3 Table) [92]. PCR analysis on genomic DNA of 293mCAT virus infected cells was performed using a combination of RT universal primer (5’ CCTACTCCGAAGACCCCTCGA-3’) and primers specific for polytropic and xenotropic ERVs (Table in S2 Table) using KOD Hotstart polymerase (Millipore, 71086) according to suggested parameters. PCR products from the reaction with RT_Universal_primer and Polytropic_JS5_rev on 293mCAT infected cells from TP^-^6, 7 and 9 mice were cloned into pCR4-TOPO vector using the TOPO TA kit following the protocol provided by the manufacturer (Invitrogen, K4575-40). Recombinant plasmids were sequenced using the T3/T7 sequencing primers from the manufacturer, and 4981_fwd and MLV_IN_T159A_fwd to determine the 5’ recombination junction. PCR with Polytropic_JS5_fwd and 7791_reverse primers determined the 3’ recombination junction for the same 293mCAT samples. PCR analysis of genomic DNA from mice tumor or thymus samples required a nested PCR. First round PCR used the RT_universal_fwd primer and the MLV_LTR_U3_rev primer. Second round of PCR used a primer pair of 7791_reverse primer and the Polytropic_JS5_ fwd primer.

### Single round of infection of K562 cells

Transfection of 293Lenti-X cells using Mirus *Trans*IT-Lenti transfection reagent with WT MLV and MLV TP^−^along with pMD2.G (Addgene) generated viruses for single round infection [93]. Viruses were quantitated using ELISA as previously described [86]. K562 cells (5x10^5^ cells) were prepared a day prior for infection in 6-well plates. For WT MLV, 500 ng of p30 was added to one well of K562 cells and for MLV IN TP^-^, 5000 ng was added. The plate was spinoculated at 1,500 g for 1 h followed by incubated for 4 h at 37°C [94]. Supernatant was removed and the cells were grown for 24 h. The cells were collected for genomic DNA extraction (see below).

### Integration target-site analysis

Genomic DNA from infected mouse tumor, thymus, 293mCAT, and K562 cells was extracted (QIAGEN #6941) and used to generate libraries for MLV integration sites. Control uninfected libraries were generated from C57BL/6J genomic DNA. Protocols for library preparation were adapted from [93]. Genomic DNA sample (5 μg) were subjected to two rounds of sonication with the following parameters: duty cycle: 5%; intensity: 3; cycles per burst: 200; time: 80 sec). Purification of DNA for the next generation library protocol used MinElute Reaction Cleanup kit (Qiagen 28204). The sonicated DNA was purified and ends of DNA fragments were repaired using End-It DNA End-Repair Kit (ER0720) as described in manufacturer’s protocol and purified after repaired DNA ends were A-tailed using Klenow Fragment (M0212S). All kits were used as described by the manufacturer’s protocol. Linker short and long strands (Table in S6 Table) were annealed by heating to 90°C and slowly cooling to room temperature in steps of 1°C per min. The annealed linkers were ligated with assigned genomic DNA sample with 3000 U of T4 ligase (M0202M) overnight at 12°C and purified. The first round of PCR used a LTR specific primer and linker specific primer (Table in S6 Table) with adapter sequence and primer binding sequence adapted from [93]. For the mouse DNA samples, the MLV_LTR_U3_rev primer (5’- GCGTTACTTAAGCTAGCTTGCCAAACCTAC-3) was used [54]. For 293mCAT and K562 cells, MLV_LTR_U5 primer (5’-CCTTGGGAGGGTCTCCTCTGAGT-3’) was used. Four PCR reactions of 100 ng DNA each were setup for each genomic DNA sample with PCR KOD Hotstart polymerase (Millipore, 71086) under these parameters: One cycle: 98°C for 2 min; 30 cycles: 98°C for 15 sec, 60°C for 30 sec, 70°C for 45 sec. The reactions were pooled and purified. The second round of PCR used a second round LTR specific primer and the same linker specific primers. These second round LTR specific primers encode a 6-nucleotide index or barcode sequence compatible for NGS, an adapter sequence for DNA clustering and a sequencing primer binding site (Table in S7 Table). Reaction parameters for the second round of PCR paralleled those of the first PCR round. All reactions were pooled and purified. Libraries were analyzed for quality and sequenced using the Illumina MiSeq system at the Molecular Biology Core Facilities at the Dana-Farber Cancer Institute. The sequences reported in this paper have been deposited in the National Center for Biotechnology Information Sequence Read Archive (Bioproject id # PRJNA548288).

### Bioinformatics

Bioinformatic analyses of integration sites were performed as described in [95]. LTR and linker sequences were cropped from 150 bp paired end reads using custom Python scripts, and the cropped reads were mapped to the reference genome (mm10 for mouse samples and hg19 for libraries from human cell lines) using HISAT2 [96]. Results were then filtered to retain high-quality alignments using SAMtools [97] and unique (deduplicate) integration sites were extracted and formatted to the browser extensible data (BED) format using custom Python scripts. Copy number of MLV integration sites from the tumor samples was calculated post filtering of high-quality alignments and prior to deduplication using custom R scripts. Copy number was calculated as described [98]. Briefly, copy number of the integrants was defined as the number of sequences having the same integration site but different breakpoints in the host DNA. Only breakpoints that were >3nt apart were counted as independent events.

Integration sites obtained from the tumor of non-infected control (NC) mouse was considered as background amplification of endogenous retroviruses obtained using this pipeline, and hence integration sites from all tumor samples overlapping with the sites from NC were computationally removed. BEDtools software suite [99] was then used to correlate unique integration sites proximal to genomic annotations such as TSS and CpG islands obtained from the University of California Santa Cruz (UCSC) database (http://genome.ucsc.edu/cgi-bin/hgTables)). Fraction of integration sites enriched at chromatin associated with Brd4 binding sites and various histone modifications (Table in S8 Table) was also computed using BEDtools suite. For ChipSeq datasets based on prior genomic versions, the coordinates were converted to the genome build used in the study (mm10 for mouse samples and hg19 for human samples) using the liftover utility from the UCSC database. Distance of integration sites from TSS, CpG islands, and Brd4 binding sites were calculated using BEDtools and histograms comparing the obtained distribution of integrants were plotted using ggplot2 [100].

Genomic annotations showing the chromatin state segmentation of K562 (wgEncodeEH000790) defined by HMM from ENCODE/BROAD was downloaded from the UCSC genome browser. Custom R scripts were written to segregate the individual chromosome state definitions from the master file and BEDtools was used to correlate integration sites within each chromosome states.

### Statistical analyses

Single factor ANOVA test was performed as described in [101] to confirm the significant changes within the experiment (*P* <0.0*5*) and Fisher’s exact test was used from computing the statistical comparisons (Table in S1 Table). Other utilized tests were as described in text.

### Amplification of MLV integrants at targeted loci

TP^-^16 integration-specific primers were designed based on the genomic location of integrants mapped using NGS (Table in S3 Table). First round PCR used forward primer at the mouse genomic sequence upstream to the integrant and MLV_LTR_U3 reverse primer using PrimeStar GXL DNA polymerase (Takara R050A) or RT_universal_fwd primer and the TP^-^16 specific reverse primer at the mouse genomic sequence downstream from the integrant using KOD HotStart polymerase. Second round PCR used the same TP^-^16 specific mouse genomic primer and either primers MLV 20R_reverse or NCAC 6327_reverse or NCAC 5166_forward. For some integrants (*Hdac6* intron 28 and near *Ccnd1)*, additional single linear amplification PCR to amplify the first round PCR was included [102]. Products were sequenced using either LTR_outside_fwd, E266K_fwd, or gene specific primers.

### Analysis of retroviral insertions overlapping H3K27ac peaks

H3K27ac ChIP-seq narrow peak files from C57BL/6 mouse thymus were obtained from ENCODE (project code ENCFF001KYC). Overlap between the H3K27ac peaks and retroviral insertion sites for each sample was assessed using the R Bioconductor packages ChIPpeakAnno and GenomicRanges. Distances between H3K27ac peaks and retroviral insertion sites were mapped using the distanceToNearest() function from the R Bioconductor package GRanges, then density plots were produced using the ggpubr and ggplot2 packages. The statistical significance of differences between distances was assessed using Wilcoxon Rank Sum tests.

## Supporting information

S1 Fig Western blot analysis of MLV -associated proteins. D17/pJET cells transfected with DNA of proviral constructs (pNCA-C) encoding WT, IN TP^-^ and IN D184N were passaged for 14 days to allow viral spread. Viral supernatants were collected, pelleted by centrifugation and analyzed western blotting using anti-SU (80S-019), anti-CA (81S-263) and anti-IN antibodies [89]. Supernatants from D17/pJET cells were used as a negative control. Positions of the protein standards are indicated at the left. Predicted MW of the WT MLV viral proteins are SU (75 kDa), CA (30 kDa) and IN (45 kDa). The two lower molecular weight products (< 30 KDa) detected by the IN antibodies are assumed to be breakdown products.
(TIF)

S2 Fig Scheme of the MLV genome and primers. Diagram of the 3’ terminal half of the MLV genome, encoding *pol* (black line), *env* (grey line) and the LTR (blue box). Individual subdomains of the IN and Env proteins are indicated: NTR [2], N-terminal region; CCD, catalytic core domain; CTD, C-terminal domain; TP, tail peptide; SU, surface; TM, transmembrane protein. Primer in blue box is an MLV universal primer located in RT, which hybridizes within a sequence conserved between known ecotropic, amphotropic, polytropic and xenotropic MLV. Primers in pink boxes are endogenous retrovirus (ERV) specific primers. M-MLV specific primers are labeled.
(TIF)

S3 Fig PCR for MLV recombinants from mice tumors and detection of mouse DNA contamination in viral infected human cell line 293mCAT. Representative agarose gels for MLV recombinant detection with universal RT primer and (A) polytropic primer (JS4) or (B) polytropic (JS5) primer. These reverse primers are located in the SU or TM regions of the Env. (C) PCR for mouse IAP. (D) PCR for mouse COX2. Mouse thymus DNA was used as positive control. Black arrows indicate expected product size.
(TIF)

S4 Fig Percentage and fold enrichment WT MLV and MLV TP^-^ within different histone modifications and Brd4 identified by ChipSeq data for K562 cells. Fold enrichment is calculated based on the frequencies of RIC at each site. Shades of blue are defined by the grid at the bottom of each panel.
(TIF)

S5 Fig MLV IN CCD and prototype foamy virus (PFV) IN CCD. (A) Sequence alignment of the PFV IN CCD and MLV IN CCD displayed using ESPript [103], with secondary structure predictions from PROMALS3D [104]. PFV IN secondary structures (red helices) are derived from the PFV intasome structure (3OS1). MLV IN secondary structures (blue helices) were assigned using the PSIPRED predicted secondary structures. (B) Homology model of the MLV IN CCD (residues 117–271) dimer was aligned using the PFV intasome structure (3OS1; [73]) [3]. Residues 266–269 (EILY) within α6 helix are in red. (C) Pseudobonds (black) between residues L268 and Y269 were predicted using UCSF Chimera [105].
(TIF)

S1 Table Fisher’s test for statistical comparison of integration profile. (DOCX)

S2 Table *MYC/Runx2* mice infected with WT MLV and MLV IN TP^-^ viruses. (DOCX)

S3 Table List of oligonucleotide primers. (DOCX)

S4 Table Percent overlap of RISs from mouse tumors and H3K27Ac peaks. (DOCX)

S5 Table Comparison of known *MYC/Runx2* CISs with MLV IN TP^-^16 tumor integrants. (DOCX)

S6 Table Linker sequence and linker specific primers. (DOCX)

S7 Table MLV LTR specific primers for second round PCR. (DOCX)

S8 Table Genomic annotations and ChipSeq datasets used in the study. (DOCX)
