# Supplementary figures and images for: Disrupting MLV integrase:BET protein interaction biases integration into quiescent chromatin and delays but does not eliminate tumor activation in a MYC/Runx2 mouse model
Source: PLoS Pathog. 2019 Dec 9;15(12):e1008154. doi: 10.1371/journal.ppat.1008154 (PMC6974304; doi:10.1371/journal.ppat.1008154)

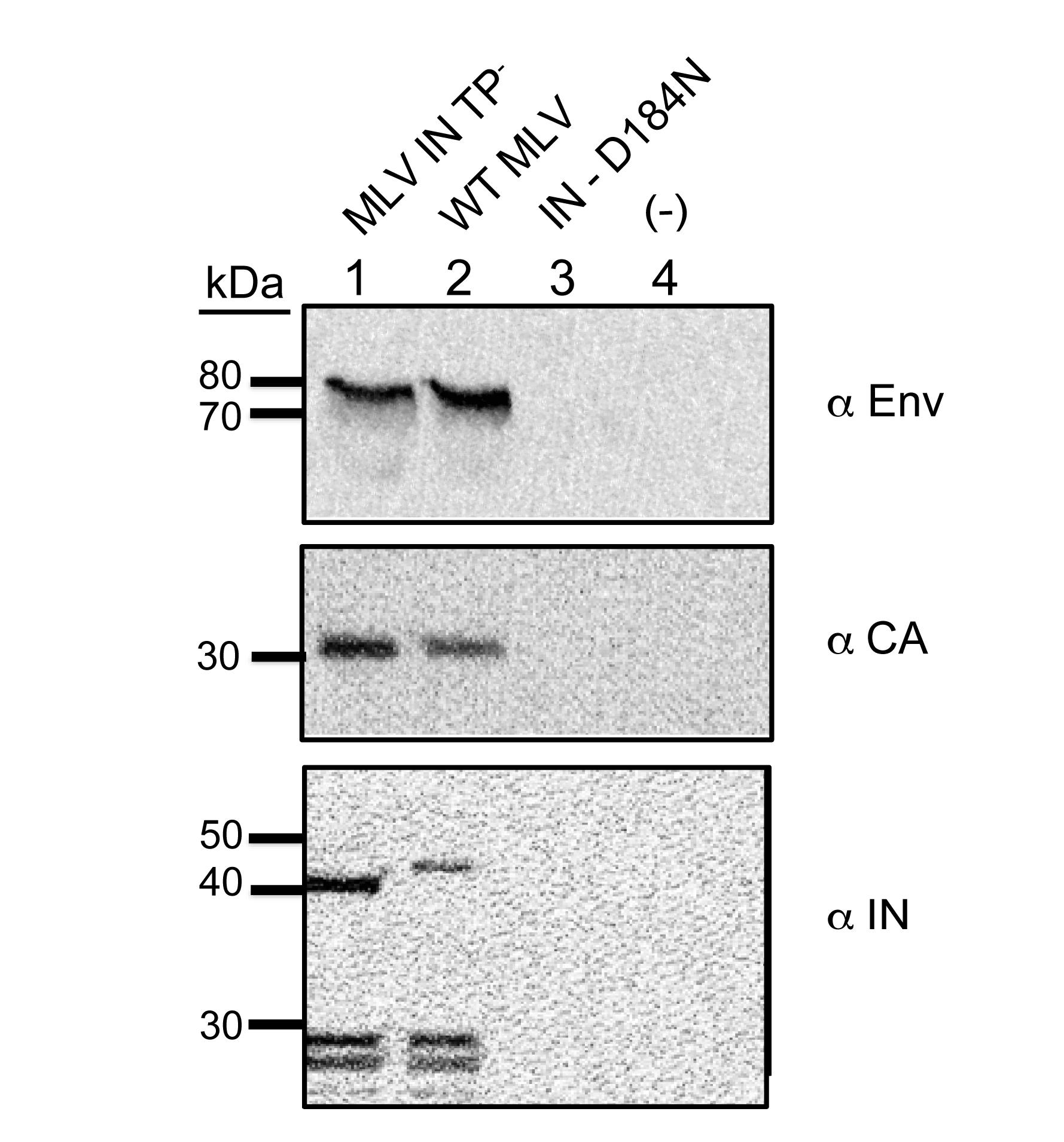

Supplement: S1 Fig — D17/pJET cells transfected with DNA of proviral constructs (pNCA-C) encoding WT, IN TP- and IN D184N were passaged for 14 days to allow viral spread. Viral supernatants were collected, pelleted by centrifugation and analyzed western blotting using anti-SU (80S-019), anti-CA (81S-263) and anti-IN antibodies [89]. Supernatants from D17/pJET cells were used as a negative control. Positions of the protein standards are indicated at the left. Predicted MW of the WT MLV viral proteins are SU (75 kDa), CA (30 kDa) and IN (45 kDa). The two lower molecular weight products (< 30 KDa) detected by the IN antibodies are assumed to be breakdown products. (TIF) [file ppat.1008154.s001.tif]

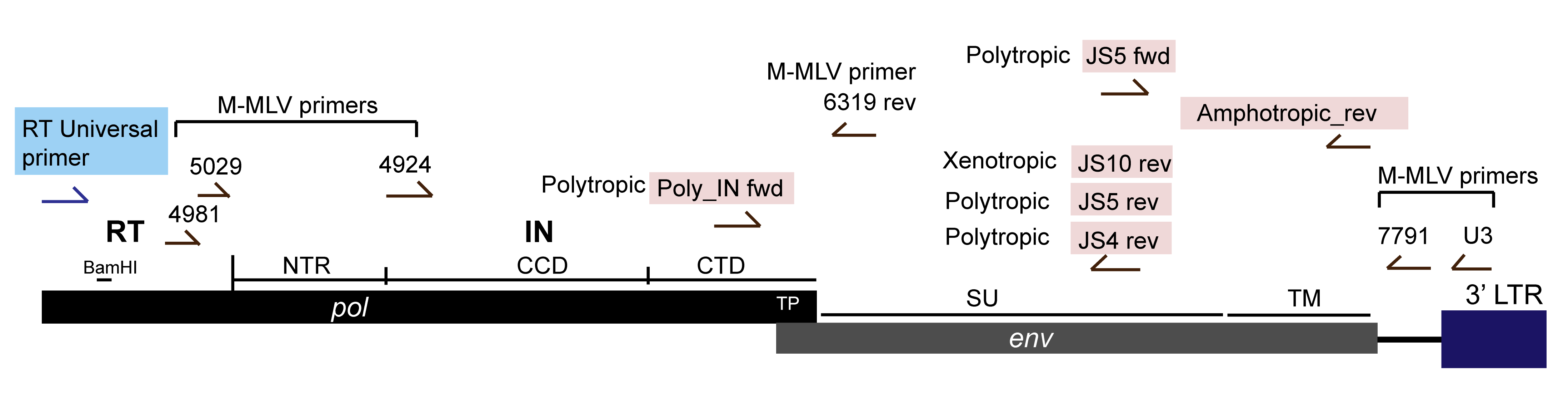

Supplement: S2 Fig — Diagram of the 3’ terminal half of the MLV genome, encoding pol (black line), env (grey line) and the LTR (blue box). Individual subdomains of the IN and Env proteins are indicated: NTR [2], N-terminal region; CCD, catalytic core domain; CTD, C-terminal domain; TP, tail peptide; SU, surface; TM, transmembrane protein. Primer in blue box is an MLV universal primer located in RT, which hybridizes within a sequence conserved between known ecotropic, amphotropic, polytropic and xenotropic MLV. Primers in pink boxes are endogenous retrovirus (ERV) specific primers. M-MLV specific primers are labeled. (TIF) [file ppat.1008154.s002.tif]

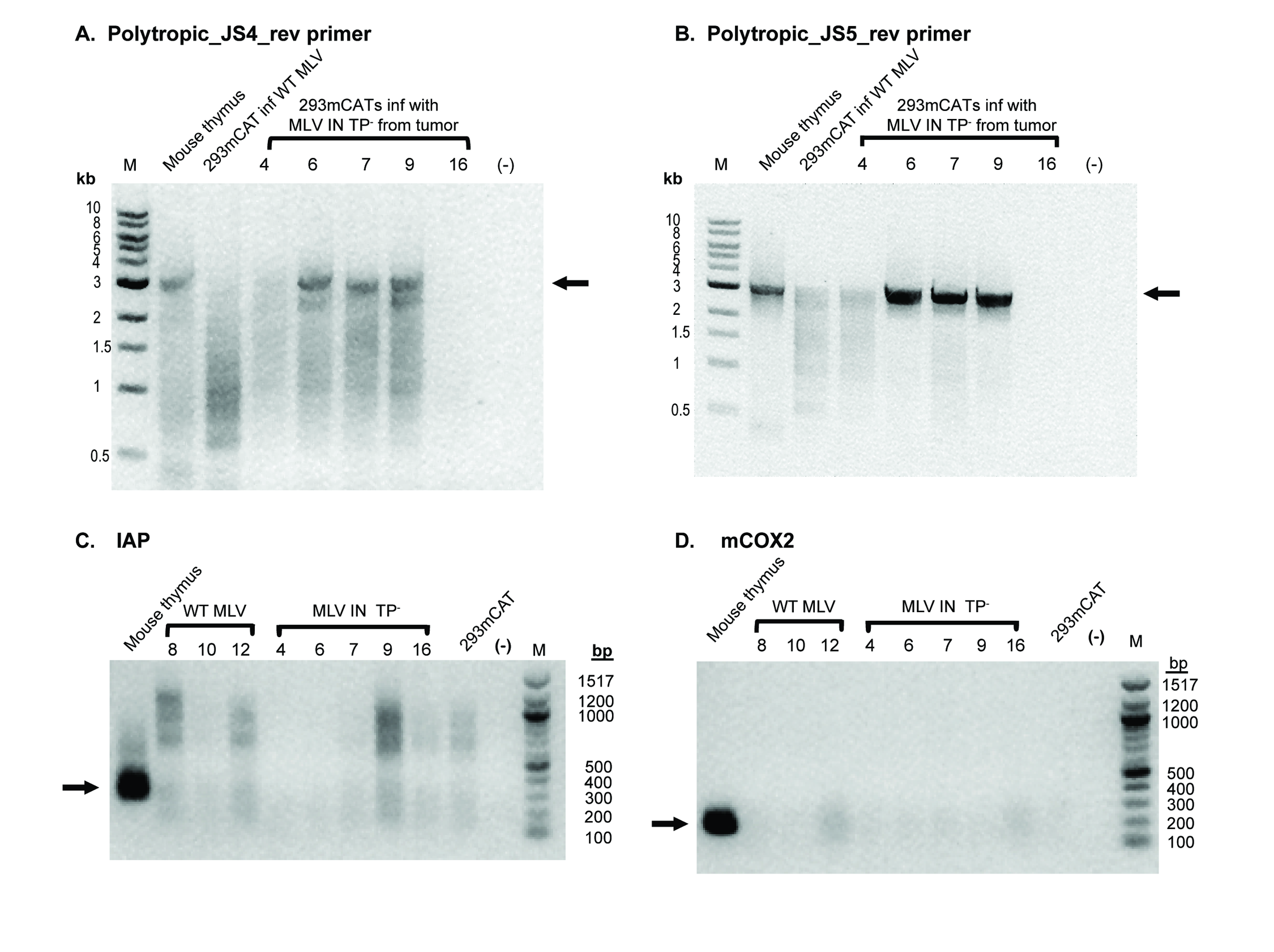

Supplement: S3 Fig — Representative agarose gels for MLV recombinant detection with universal RT primer and (A) polytropic primer (JS4) or (B) polytropic (JS5) primer. These reverse primers are located in the SU or TM regions of the Env. (C) PCR for mouse IAP. (D) PCR for mouse COX2. Mouse thymus DNA was used as positive control. Black arrows indicate expected product size. (TIF) [file ppat.1008154.s003.tif]

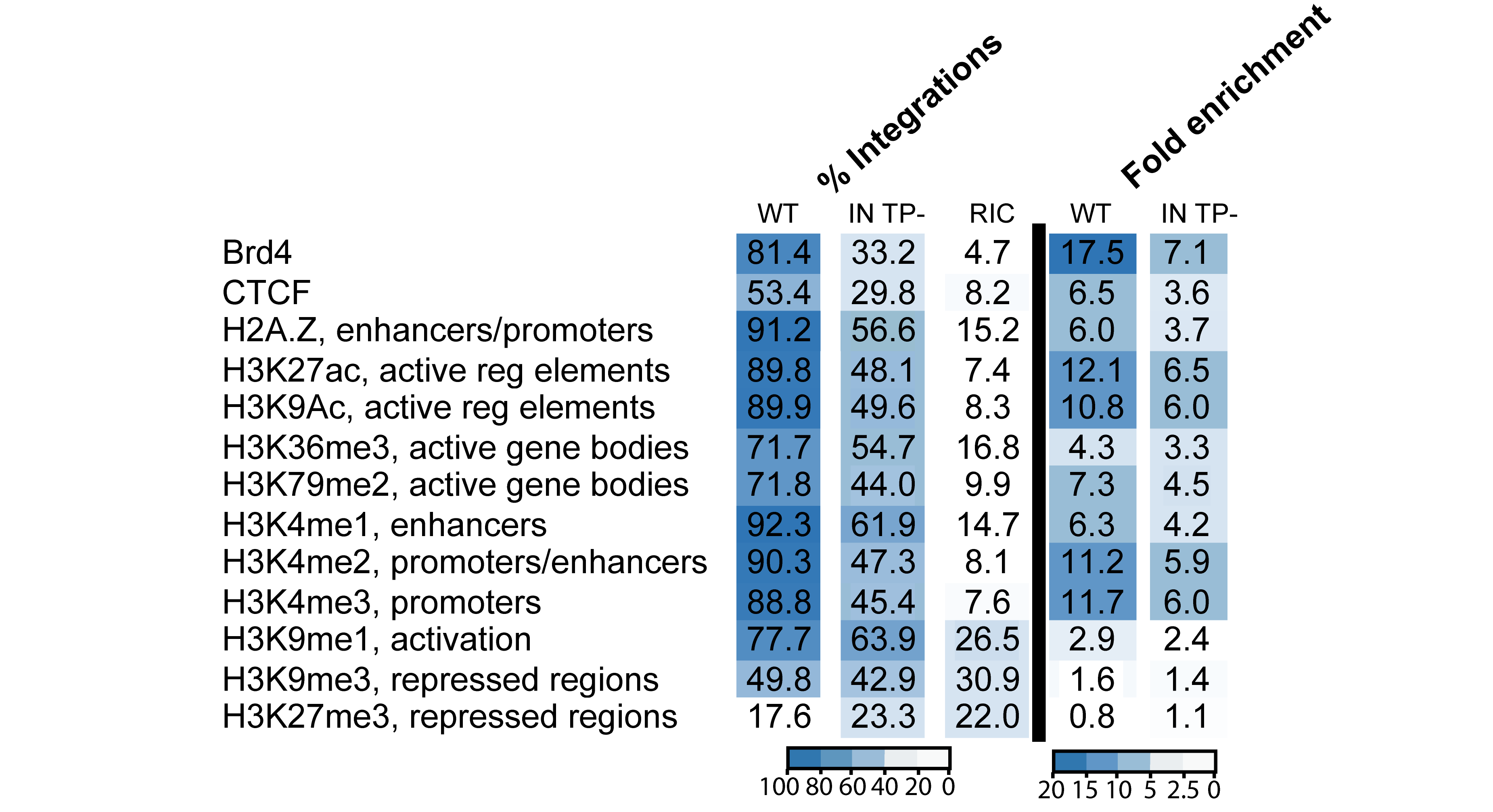

Supplement: S4 Fig — Fold enrichment is calculated based on the frequencies of RIC at each site. Shades of blue are defined by the grid at the bottom of each panel. (TIF) [file ppat.1008154.s004.tif]

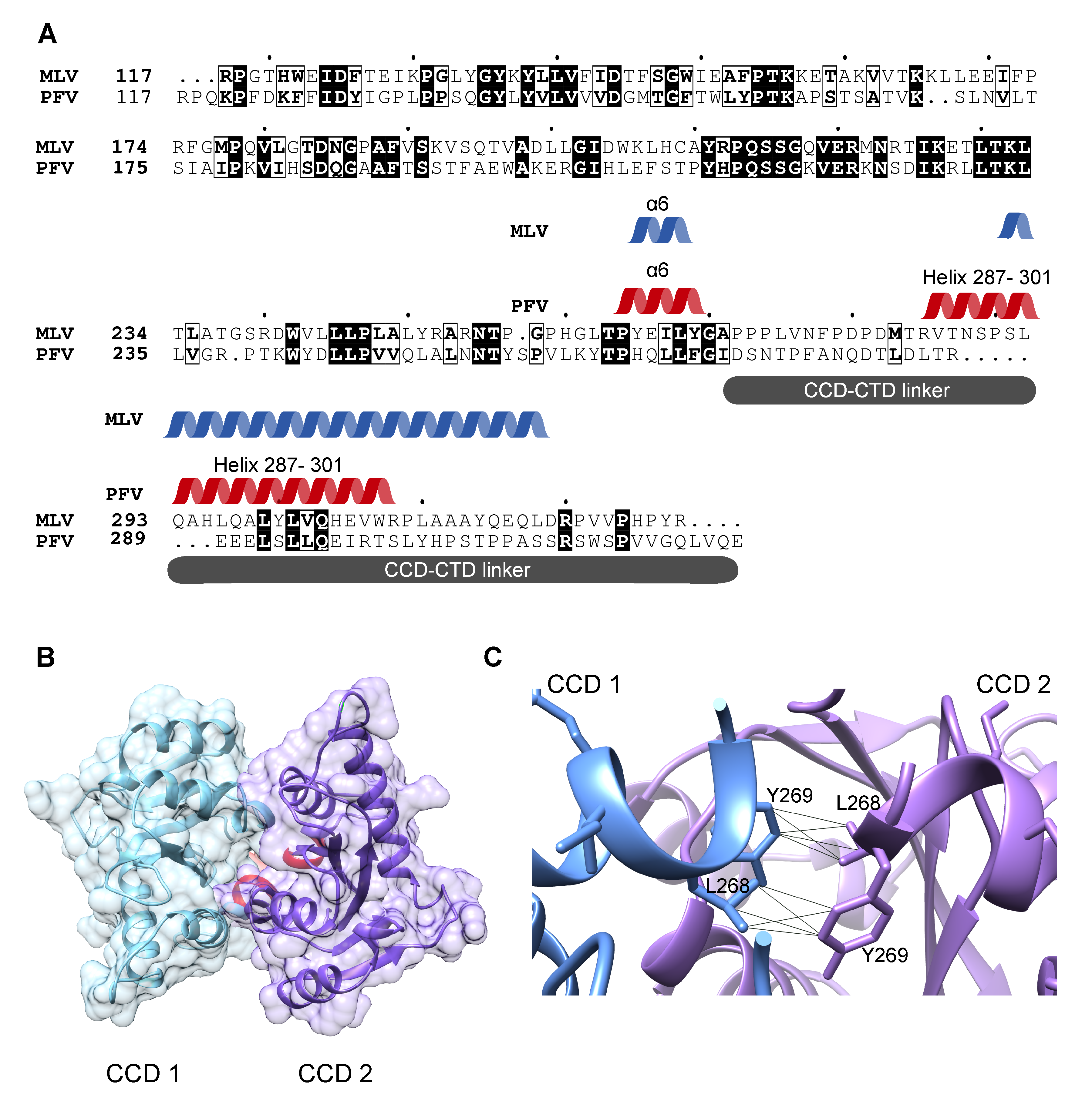

Supplement: S5 Fig — (A) Sequence alignment of the PFV IN CCD and MLV IN CCD displayed using ESPript [103], with secondary structure predictions from PROMALS3D [104]. PFV IN secondary structures (red helices) are derived from the PFV intasome structure (3OS1). MLV IN secondary structures (blue helices) were assigned using the PSIPRED predicted secondary structures. (B) Homology model of the MLV IN CCD (residues 117–271) dimer was aligned using the PFV intasome structure (3OS1; [73]) [3]. Residues 266–269 (EILY) within α6 helix are in red. (C) Pseudobonds (black) between residues L268 and Y269 were predicted using UCSF Chimera [105]. (TIF) [file ppat.1008154.s005.tif]
