# Supplementary material for: Disrupting MLV integrase:BET protein interaction biases integration into quiescent chromatin and delays but does not eliminate tumor activation in a MYC/Runx2 mouse model
Source: PLoS Pathog. 2019 Dec 9;15(12):e1008154. doi: 10.1371/journal.ppat.1008154 (PMC6974304; doi:10.1371/journal.ppat.1008154)
Supplement: S1 Table — (DOCX) [file ppat.1008154.s006.docx]

**S1 Table. Fisher’s test for statistical comparison of integration profile.**

**Mouse tumors from *MYC/Runx2* mice model^a^**

| **TSS +/- 1 kb** |  | NC | WT6 | WT8 | WT10 | WT12 | TP^-^4 | TP^-^ 6 | | TP^-^ 7 | TP^-^9 | | TP^-^16 | |  |  |  |
| --- | --- | --- | --- | --- | --- | --- | --- | --- | --- | --- | --- | --- | --- | --- | --- | --- | --- |
|  | NC |  | 2.50E-65 | 1.38E-44 | 9.02E-47 | 1.14E-32 | 3.66E-47 | | 9.42E-32 | 1.49E-46 | | 1.48E-25 | | 2.51E-08 |  |  |  |
|  |  |  |  |  |  |  |  | |  |  | |  |  | |  |  |  |
| **CpG +/- 1 kb** |  | NC | WT6 | WT8 | WT10 | WT12 | TP^-^4 | | TP^-^ 6 | TP^-^ 7 | | TP^-^9 | TP^-^16 | |  |  |  |
|  | NC |  | 4.44E-61 | 6.23E-40 | 1.00E-43 | 2.12E-38 | 3.66E-47 | | 9.42E-32 | 1.49E-46 | | 1.48E-25 | 3.66E-47 | |  |  |  |

| **TSS +/- 1 kb** |  | TP^-^16 |
| --- | --- | --- |
|  | WT6 | 4.39E-06 |
|  |  |  |
| **CpG +/- 1 kb** |  | TP^-^16 |
|  | WT6 | **0.0001** |

| **Brd4 +/- 1 kb** |  | NC | WT6 | TP^-^16 |
| --- | --- | --- | --- | --- |
|  | NC |  | 1.86E-226 | 2.89E-08 |
|  | WT6 |  |  | 1.51E-37 |

| **H3K4me1 +/- 1 kb** |  | NC | WT6 | TP^-^16 |
| --- | --- | --- | --- | --- |
|  | NC |  | < 2.2E-299 | 8.00E-40 |
|  | WT6 |  |  | < 2.2E-299 |

| **H3K4me3 +/- 1 kb** |  | NC | WT6 | TP^-^16 |
| --- | --- | --- | --- | --- |
|  | NC |  | 2.65E-212 | 1.23E-19 |
|  | WT6 |  |  | 2.25E-20 |

**K562 infected cells^b^**

| **TSS** |  | RIC | WT | IN TP^-^ |
| --- | --- | --- | --- | --- |
|  | RIC | 1 | < 2.2 x10-299 | 7.42E-20 |
|  | **WT** |  |  | 5.65E-44 |
|  |  |  |  |  |
| **CpG** |  | RIC | WT | IN TP^-^ |
|  | RIC | 1 | < 2.2 x10-299 | 1.16E-25 |
|  | WT |  |  | 3.13E-47 |

**K562 infected cells (15-chromatin states)**^b^

| **State 11** |  | RIC | WT | IN TP^-^ |
| --- | --- | --- | --- | --- |
|  | RIC |  | 6.63E-40 | 4.03E-15 |
|  | WT |  |  | 2.08E-50 |
|  |  |  |  |  |
| **State 13** |  | RIC | WT | IN TP^-^ |
|  | RIC |  | < 2.2 x10-299 | 1.71E-119 |
|  | WT |  |  | 2.92E-61 |

^a^P-values are shown for NC vs. tumor samples, tumor WT6 vs. TP^-^16

^b^P-values for infected K562 samples are shown for RIC vs. infected K562 cells and WT vs IN TP^-^
