# Supplementary material for: Disrupting MLV integrase:BET protein interaction biases integration into quiescent chromatin and delays but does not eliminate tumor activation in a MYC/Runx2 mouse model
Source: PLoS Pathog. 2019 Dec 9;15(12):e1008154. doi: 10.1371/journal.ppat.1008154 (PMC6974304; doi:10.1371/journal.ppat.1008154)
Supplement: S2 Table — (DOCX) [file ppat.1008154.s007.docx]

**S2 Table. *MYC/Runx2* mice infected with WT MLV and MLV IN TP^-^ viruses**

|  | **ID** | **Sex** | **DoD** |  | **ID** | **Sex** | **DoD** |
| --- | --- | --- | --- | --- | --- | --- | --- |
| **WT MLV** | Gim16(WT) inf-1 | F | 34 | **MLV IN TP^-^** | Gim16(TP-)inf-1 | M | 48 |
|  | Gim16(WT) inf-2 | F | 34 |  | Gim16(TP-)inf-2 | M | 48 |
|  | Gim16(WT) inf-3 | F | 34 |  | Gim16(TP-)inf-3 | F | 48 |
|  | Gim16(WT) inf-4 | M | 34 |  | Gim16(TP-)inf-4^a,b^ | M | 50 |
|  | Gim16(WT) inf-5 | M | 34 |  | Gim16(TP-)inf-5 | M | 56 |
|  | Gim16(WT) inf-6 ^a^ | M | 34 |  | Gim16(TP-)inf-6^a,b^ | F | 57 |
|  | Gim16(WT) inf-7 | M | 34 |  | Gim16(TP-)inf-7 ^a,b^ | F | 37 |
|  | Gim16(WT) inf-8 ^a^ | F | 36 |  | Gim16(TP-)inf-8 | M | 59 |
|  | Gim16(WT) inf-9 | F | 52 |  | Gim16(TP-)inf-9 ^a,b^ | F | 63 |
|  | Gim16(WT) inf-10 ^a^ | F | 52 |  | Gim16(TP-)inf-10 | M | 63 |
|  | Gim16(WT) inf-11 | M | 52 |  | Gim16(TP-)inf-11 | F | 63 |
|  | Gim16(WT) inf-12 ^a^ | M | 30 |  | Gim16(TP-)inf-12 ^b^ | M | 28 |
|  | Gim16(WT) inf-13 | F | 63 |  | Gim16(TP-)inf-13 ^b^ | M | 30 |
|  | Gim16(WT) inf-14 | F | 63 |  | Gim16(TP-)inf-14 | M | 69 |
|  | Gim16(WT) inf-15 | F | 41 |  | Gim16(TP-)inf-15 ^b^ | M | 33 |
|  | Gim16(WT) inf-16 | M | 41 |  | Gim16(TP-)inf-16 ^a,b^ | F | 34 |
|  | Gim16(WT) inf-17 | M | 64 |  | Gim16(TP-)inf-17 ^b^ | M | 34 |
|  | Gim16(WT) inf-18 | F | 56 |  | Gim16(TP-)inf-18 ^b^ | M | 36 |
|  | Gim16(WT) inf-19 | F | 56 |  | Gim16(TP-)inf-19 ^b^ | M | 36 |
|  | Gim16(WT) inf-20 | F | 34 |  | Gim16(TP-)inf-20 | M | 65 |
|  | Gim16(WT) inf-21 | F | 34 |  | Gim16(TP-)inf-21 | F | 70 |
|  | Gim16(WT) inf-22 | F | 34 |  | Gim16(TP-)inf-22 | M | 70 |
|  | Gim16(WT) inf-23 | F | 34 |  | Gim16(TP-)inf-23 | M | 72 |
|  | Gim16(WT) inf-24 | F | 35 |  |  |  |  |
|  | Gim16(WT) inf-25 | F | 35 |  |  |  |  |
|  | Gim16(WT) inf-26 | F | 35 |  |  |  |  |
|  | Gim16(WT) inf-27 | M | 35 |  |  |  |  |
|  | Gim16(WT) inf-28 | F | 41 |  |  |  |  |
|  | Gim16(WT) inf- | F | 23 |  |  |  |  |

^a^DNA from tumors from these mice were analyzed by next-generation sequencing

^b^Viral integrants analyzed for recombination with ERV
