# Supplementary material for: Disrupting MLV integrase:BET protein interaction biases integration into quiescent chromatin and delays but does not eliminate tumor activation in a MYC/Runx2 mouse model
Source: PLoS Pathog. 2019 Dec 9;15(12):e1008154. doi: 10.1371/journal.ppat.1008154 (PMC6974304; doi:10.1371/journal.ppat.1008154)
Supplement: S3 Table — (DOCX) [file ppat.1008154.s008.docx]

**S3 Table. List of oligonucleotide primers**

| Primer | Primer Sequence (5' ->3’) | Reference^a^ |
| --- | --- | --- |
| Virus class specific primers |  |  |
| Polytropic_JS4_rev | GCAGCCTCTATACAACCTGGGACGGGAG | [92] |
| Polytropic_JS5_rev | GCAGCCTCTATACTCCCTGAGACTGCCC | [92] |
| Polytropic_JS5_fwd | GCAGCCTCTATACTCCCTGAGACTGCCC |  |
| Polytropic_JS6_rev | ACGGTCTCTATGGTACCTGGGGCTCCCC | [92] |
| Polytropic_IN_fwd | TAAAAGCGGCGACAACCCCTCC |  |
| Xenotropic_JS10_rev | ACGGTCTCTATGGTGCCTGGGGCTCCCC | [92] |
| Amphotropic _rev | ATTCATGGCTCGTACTCTATGGGTTTTAGC |  |
| RT_universal_ fwd | CCTACTCCGAAGACCCCTCGA |  |
|  |  |  |
| Moloney MLV primers |  |  |
| MLV_LTR_U3_rev | GCGTTACTTAAGCTAGCTTGCCAAACCTAC |  |
| MLV_LTR_U5_rev | CCTTGGGAGGGTCTCCTCTGAGT |  |
| MLV_IN_T159A_fwd | CTTCCCAACCAAGAAAGAAGCCGCCAAGGTCGTAACCAAGAAG |  |
| 4981_fwd | GGCTAGAGGCAACCGGATGG |  |
| 7791_rev | ccttaaggCCCCCCTTTTTCTGGAGACTAAATA^b^ | (6320;[45]) |
| 4924_fwd | gatatacatatgGCCGTTAAACAGGGA^c^ | (3807;[45]) |
| 6319_rev | AGTACTGCTTCGCCCGGCTCCAGTCCTCA |  |
| 5166_fwd | CACAGAGACTCCAGACACCTCTACC |  |
| 20R | GCCCAGACTGGGATTACACCACC |  |
|  |  |  |
| TP^-^16 integrants-specific primers |  |  |
| HDAC6_ex3_fwd | TCCAGTACCAACTGGCACTCTTGT |  |
| HDAC6_ex3_rev | ATTCTTCTACTAGACAGCGAAAGAGTAGGC |  |
| HDAC6_intr28_rev | ATATGGTCTGCCACCATGAAGCCTCTGAA |  |
| MAPK13_inr_rev | GTGAAACAGGTCAGGGTCAGCAA |  |
| RasGRP1_rev | ACATTGGTCCTTTGCAGCTTT |  |
| CCND1_rev | AAATTAGGAAGGAGCCTATCGTGT |  |
| GNG7_intr_rev | GTCACGGTGCTGTAGGTCATA |  |
| LTR_outside_fwd | GGCAAGCTAGCTTAAGTAACGCC |  |
| NCAC_6327_rev | AGCCGGGCGAAGCAGTACT |  |
|  |  |  |
| TP^-^ Gene block sequence | GACCATCCTTTGCGGCCGCTAACTGACATGGCCCGGAGCACCCTGAGCAAGCCTCTTAAGAACAAAGTGAATCCCCGGGGACCTCTGATCCCCTTAATTCTTCTGATGCTCAGAGGGGTCAGTACTGCTTCGCCCGG |  |
|  |  |  |
| TP^-^ gene block cloning primers |  |  |
| NCACXN_ScaI6330_rev | CCGGGCGAAGCAGTACTGA |  |
| NCACXN_NotI6220_fwd | GACCATCCTTTGCGGCCG |  |
| NCAC_8290_rev | GGCGTTACTTAAGCTAGCTTGCC |  |
| NCACXN_6327_fwd | AGTACTGCTTCGCCCGGCT |  |
|  |  |  |
| Mouse DNA primers |  |  |
| Mouse_mt_COX2_fwd | TTCTACCAGCTGTAATCCTTA | [91] |
| Mouse_mt_COX2_rev | GTTTTAGGTCGTTTGTTGGGAT | [91] |
| Mouse_IAP_fwd | ATAATCTGCGCATGAGCCAAGG | [90] |
| Mouse_IAP_rev | AGGAAGAACACCACAGACCAG | [90] |
|  |  |  |
| CCD Mutants primers |  |  |
| 102510NdeIINteinIN forward | ggaattccatatgATAGAAAATTCATCACCCTACACCTCAG |  |
| 102510XhoIInteinIN1-407 reverse | ccggctcgaGGGCCTCGCGGGTTAACC |  |
| E266A_fwd | CCCATGGCCTCACCCCATATGCCATCTTATATGGGGCACCCCCGCC |  |
| E266A_rev | GGCGGGGGTGCCCCATATAAGATGGCATATGGGGTGAGGCCATGGG |  |
| E266K_fwd | CCCATGGCCTCACCCCATATAAGATCTTATATGGGGCACCCCCGCC |  |
| L268A_fwd | CATGGCCTCACCCCATATGAGATCGCCTATGGGGCACCCCCG |  |
| L268A_rev | GCGGGGGTGCCCCATAGGCGATCTCATATGGGGTGAGGCCAT |  |
| Y269A_fwd | GCCTCACCCCATATGAGATCTTAGCCGGGGCACCCCCGC |  |
| Y269A_rev | GCGGGGGTGCCCCGGCTAAGATCTCATATGGGGTGAGGC |  |

^a^All oligonucleotides without a reference were generated for this manuscript

^b^Described as primer 6320 in [43]; lower case letters are not encoded by virus

^c^Described as primer 3807 in [43]; lower case letters are not encoded by virus
