# Supplementary material for: Disrupting MLV integrase:BET protein interaction biases integration into quiescent chromatin and delays but does not eliminate tumor activation in a MYC/Runx2 mouse model
Source: PLoS Pathog. 2019 Dec 9;15(12):e1008154. doi: 10.1371/journal.ppat.1008154 (PMC6974304; doi:10.1371/journal.ppat.1008154)
Supplement: S4 Table — (DOCX) [file ppat.1008154.s009.docx]

**S4 Table. Percent overlap of RISs from mouse tumors and H3K27Ac peaks**

| **Samples** | **Unique sites** | **Total H3K27Ac peaks** | **Number of sites overlapping with H3K27Ac peaks** | **% Unique sites overlapping with H3K27Ac peaks** |
| --- | --- | --- | --- | --- |
| WT6 | 14049 | 34687 | 4414 | 31.4 |
| WT8 | 4319 | 34687 | 1348 | 31.2 |
| WT10 | 5043 | 34687 | 1779 | 35.3 |
| WT12 | 1422 | 34687 | 528 | 37.1 |
| TP^-^4 | 3366 | 34687 | 1125 | 33.4 |
| TP^-^6 | 2370 | 34687 | 724 | 30.5 |
| TP^-^7 | 7544 | 34687 | 1808 | 24.0 |
| TP^-^9 | 1601 | 34687 | 427 | 26.7 |
| TP^-^16 | 579 | 34687 | 74 | 12.8 |
