# Supplementary material for: Disrupting MLV integrase:BET protein interaction biases integration into quiescent chromatin and delays but does not eliminate tumor activation in a MYC/Runx2 mouse model
Source: PLoS Pathog. 2019 Dec 9;15(12):e1008154. doi: 10.1371/journal.ppat.1008154 (PMC6974304; doi:10.1371/journal.ppat.1008154)
Supplement: S5 Table — (DOCX) [file ppat.1008154.s010.docx]

**S5 Table. Comparison of known *MYC/Runx2*CISs with MLV IN TP^-^16 tumor integrants**

| Gene | Integrant position | | | Integrant copy #  (rank) ^a^ | CIS (rank^c^) |
| --- | --- | --- | --- | --- | --- |
| *Rasgrp1* | chr2 | 117,341,395 | intergenic | 4 | 1 |
|  |  | 117,410,421 | intergenic | 9 |  |
|  |  | 117,419,644 | intergenic | 4 |  |
|  |  | 117,434,636 | intergenic | 28 (3-4) ^b^ |  |
| Jdp2 | chr12 | 85,597,389 | intergenic | 3 | 2 |
|  |  | 85,641,374 | intergenic | 18 |  |
| *Hdac6* | chrX | 7,930,719 | intron 28 | 26 (5-7) ^b^ | 5 |
|  |  | 7,931,586 | exon 25 | 6 |  |
|  |  | 7,941,877 | exon 10 | 4 |  |
|  |  | 7,944,693 | intron 5 | 33 (1) |  |
|  |  | 7,946,933 | exon 3 | 26 (5-7) ^b^ |  |
| *Hdac6/Otud5/Pim2* | chrX | 7,918,395 | intergenic | 5 | 5 |
| Otud5 | chrX | 7,864,391 | intron 2 | 2 | 5 |
|  |  | 7,868,169 | intron 5 | 2 |  |
| Ahi1 | chr10 | 20,991,590 | intron 15 | 2 | 6 |
|  |  | 21,048,422 | intron 19 | 2 |  |
|  |  | 21,055,250 | intron 20 | 23 (8-9) |  |
| Ahi1/Myb | chr10 | 21,080,468 | intergenic | 6 | 6 |
|  |  | 21,117,776 | intergenic | 2 |  |
| Zmiz1 | chr14 | 25,320,612 | intergenic | 7 | 7 |
|  |  | 25,608,859 | intron 6 | 3 |  |
| *Ccnd1* | chr7 | 144,940,638 | intergenic | 28 (3-5) ^b^ | 8 |
|  |  | 144,964,351 | intergenic | 4 |  |
|  |  | 144,996,507 | intergenic | 2 |  |
| Otx2 | chr14 | 4,8741,287 | intergenic | 6 | 9 |
| *Pik3r5* | chr11 | 6,8434,859 | intron 1 | 3 | 10 |
|  |  | 6,8456,063 | intron 1 | 4 |  |
|  |  | 6,8461,892 | intron 1 | 2 |  |
| *Bcl9I* | chr9 | 44,486,500 | intron 1 | 2 | 14 |
|  |  | 44,488,249 | intron 1 | 4 |  |
|  |  | 44,489,540 | intron 1 | 3 |  |
| *Pim1* | chr17 | 29,495,444 | exon 6 | 19 | 22 |
| *Myo16* | chr8 | 10,157,125 | intron 1 | 22 (10) | 23 |
| *MapK13* | chr17 | 28,773,031 | intron 3 | 31 (2) | N/A |
| *Gng7* | chr10 | 80,957,684 | intron 2 | 28 (3-4) | N/A |
| *Seh1* | chr18 | 67,775,172 | intron 1 | 23 (8-9) | N/A |

^a^Maximum copy number of integrants from MLV IN TP^-^16 tumors in this analysis=33;

rank based on the top 10 copy number genes; range indicates multiple genes with identical copy numbers.

^b^Integrant analyzed for IN TP^-^ genotype

^c^ Based on Huser et al, Fig 1E [20]; N/A not applicable
