# Supplementary material for: Disrupting MLV integrase:BET protein interaction biases integration into quiescent chromatin and delays but does not eliminate tumor activation in a MYC/Runx2 mouse model
Source: PLoS Pathog. 2019 Dec 9;15(12):e1008154. doi: 10.1371/journal.ppat.1008154 (PMC6974304; doi:10.1371/journal.ppat.1008154)
Supplement: S6 Table — (DOCX) [file ppat.1008154.s011.docx]

S6 Table. Linker sequence and linker specific primers

| **Usage** | **Sequence**^a^ |
| --- | --- |
| Linker 1 short strand | 5'-PO_4_-GTCCCTTAAGCGGAG-NH_2_-3' |
| Linker 1 long strand | **GTAATACGACTCACTATAGGGC**CTCCGCTTAAGGGACT |
| Linker 2 short strand | 5'-PO_4_-CGAGGCGTCTAATGC-NH_2_-3' |
| Linker 2 long strand | **GCTATAGCAGCACATCAGTTAG**GCATTAGACGCCTCGT |
| Linker 3 short strand | 5'-PO_4_-CTATGACGGTGACGC-NH_2_-3' |
| Linker 3 long strand | **GAGAATCCATGAGTATGCTCAC**GCGTCACCGTCATAGT |
| Linker 5 short strand | 5'-PO_4_-CTGAGACGTCGATGC-NH_2_-3' |
| Linker 5 long strand | **GATCATGCGAGATACATCTCAG**GCATCGACGTCTCAGT |
| Linker 6 short strand | 5'-PO_4_-CGATGCGGTAACTGC-NH_2_-3' |
| Linker 6 long strand | **GTATCTCAACAAGCAGCTTGAG**GCAGTTACCGCATCGT |
| Linker 7 short strand | 5'-PO_4_-CTAGTACGGAGTCGC-NH_2_-3' |
| Linker 7 long strand | **GCCATGGAATATGCAATCTGAC**GCGACTCCGTACTAGT |
| Linker 8 short strand | 5'-PO_4_- CGGTGAGCGCATATC-NH_2_-3' |
| Linker 8 long strand | **CAACTTGCGTGCAATTAACGAG**GATATGCGCTCACCGT |
| Linker 9 short strand | 5'-PO_4_- ACGTAGGTGCGCATC-NH_2_-3' |
| Linker 9 long strand | **CAGGATGCGTAATACGAATCTC**GATGCGCACCTACGTT |
| Linker 10 short strand | 5'-PO_4_-CCGGTCAGCATAGTG-NH_2_-3' |
| Linker 10 long strand | **GACTTGAACCGTAGCATCTAAG**CACTATGCTGACCGGT |
| Linker 11 short strand | 5'-PO_4_- CTGATACCGGCGTAG-NH_2_-3' |
| Linker 11 long strand | **GAGCCTACGTTACGCAATATAG**CTACGCCGGTATCAGT |
| **Linker specific Primers** | **Sequence^b^** |
| Linker 1 | *CAAGCAGAAGACGGCATACGAGAT*CGGTCTCGGCATTCCTGCTGAACCGCTCTTCCGATCT**GTAATACGACTCACTATAGGGC** |
| Linker 2 | *CAAGCAGAAGACGGCATACGAGAT*CGGTCTCGGCATTCCTGCTGAACCGCTCTTCCGATCT**GCTATAGCAGCACATCAGTTAG** |
| Linker 3 | *CAAGCAGAAGACGGCATACGAGAT*CGGTCTCGGCATTCCTGCTGAACCGCTCTTCCGATCT**GAGAATCCATGAGTATGCTCAC** |
| Linker 5 | *CAAGCAGAAGACGGCATACGAGAT*CGGTCTCGGCATTCCTGCTGAACCGCTCTTCCGATCT**GATCATGCGAGATACATCTCAG** |
| Linker 6 | *CAAGCAGAAGACGGCATACGAGAT*CGGTCTCGGCATTCCTGCTGAACCGCTCTTCCGATCT**GTATCTCAACAAGCAGCTTGAG** |
| Linker 7 | *CAAGCAGAAGACGGCATACGAGAT*CGGTCTCGGCATTCCTGCTGAACCGCTCTTCCGATCT**GCCATGGAATATGCAATCTGAC** |
| Linker 8 | *CAAGCAGAAGACGGCATACGAGAT*CGGTCTCGGCATTCCTGCTGAACCGCTCTTCCGATCT**CAACTTGCGTGCAATTAACGAG** |
| Linker 9 | *CAAGCAGAAGACGGCATACGAGAT*CGGTCTCGGCATTCCTGCTGAACCGCTCTTCCGATCT**CAGGATGCGTAATACGAATCTC** |
| Linker 10 | *CAAGCAGAAGACGGCATACGAGAT*CGGTCTCGGCATTCCTGCTGAACCGCTCTTCCGATCT**GACTTGAACCGTAGCATCTAAG** |
| Linker 11 | *CAAGCAGAAGACGGCATACGAGAT*CGGTCTCGGCATTCCTGCTGAACCGCTCTTCCGATCT**GAGCCTACGTTACGCAATATAG** |

^a^Oligonucleotides are modified with 5’ phosphate end and 3’ amino modifier (Integrated Data Technology); linker (bold).

^b^Linker specific primers contain an adapter sequence (italics), primer binding sequence (underlined), and linker (bold).
