# Supplementary material for: Disrupting MLV integrase:BET protein interaction biases integration into quiescent chromatin and delays but does not eliminate tumor activation in a MYC/Runx2 mouse model
Source: PLoS Pathog. 2019 Dec 9;15(12):e1008154. doi: 10.1371/journal.ppat.1008154 (PMC6974304; doi:10.1371/journal.ppat.1008154)
Supplement: S7 Table — (DOCX) [file ppat.1008154.s012.docx]

S7 Table. MLV LTR specific primers for second round PCR

| MLV U3 LTR second round PCR^a^ | | |
| --- | --- | --- |
| MLV U3 LTR Index Primer - ACTTGA | *AATGATACGGCGACCACCGAGATCTACAC*TCTTTCCCTACACGACGCTCTTCCGATCT**ACTTGA**CCAAACCTACAGGTGGGGTCTTTC | |
| MLV U3 LTR Index Primer - GATCAG | *AATGATACGGCGACCACCGAGATCTACA*CTCTTTCCCTACACGACGCTCTTCCGATCT**GATCAG**CCAAACCTACAGGTGGGGTCTTTC | |
| MLV U3 LTR Index Primer - GGCTAC | *AATGATACGGCGACCACCGAGATCTACAC*TCTTTCCCTACACGACGCTCTTCCGATCT**GGCTAC**CCAAACCTACAGGTGGGGTCTTTC | |
| MLV U3 LTR Index Primer - TAGCTT | *AATGATACGGCGACCACCGAGATCTACAC*TCTTTCCCTACACGACGCTCTTCCGATCT**TAGCTT**CCAAACCTACAGGTGGGGTCTTTC | |
| MLV U3 LTR Index primer - GTGGCC | *AATGATACGGCGACCACCGAGATCTACAC*TCTTTCCCTACACGACGCTCTTCCGATCT**GTGGCC**CCAAACCTACAGGTGGGGTCTTTC | |
| MLV U3 LTR Index primer- CGTACG | *AATGATACGGCGACCACCGAGATCTACAC*TCTTTCCCTACACGACGCTCTTCCGATCT**CGTACG**CCAAACCTACAGGTGGGGTCTTTC | |
| MLV U3 LTR Index primer - GAGTGG | *AATGATACGGCGACCACCGAGATCTACAC*TCTTTCCCTACACGACGCTCTTCCGATCT**GAGTGG**CCAAACCTACAGGTGGGGTCTTTC | |
| MLV U3 LTR Index primer - ACTGAT | *AATGATACGGCGACCACCGAGATCTACAC*TCTTTCCCTACACGACGCTCTTCCGATCT**ACTGAT**CCAAACCTACAGGTGGGGTCTTTC | |
| MLV U3 LTR Index primer - CTTGTA | *AATGATACGGCGACCACCGAGATCTACAC*TCTTTCCCTACACGACGCTCTTCCGATCT**CTTGTA**CCAAACCTACAGGTGGGGTCTTTC | |
| MLV U3 LTR Index primer - ATTCCT | *AATGATACGGCGACCACCGAGATCTACAC*TCTTTCCCTACACGACGCTCTTCCGATCT**ATTCCT**CCAAACCTACAGGTGGGGTCTTTC | |
| MLV U5 LTR second round PCR^b^ | | |
| MLV U5 LTR Index Primer - ACTTGA | | *AATGATACGGCGACCACCGAGATCTACAC*TCTTTCCCTACACGACGCTCTTCCGATCT**ACTTGA**TGACTACCCGTCAGCGGGGGTC |
| MLV U5 LTR Index Primer - GATCAG | | *AATGATACGGCGACCACCGAGATCTACAC*TCTTTCCCTACACGACGCTCTTCCGATCT**GATCAG**TGACTACCCGTCAGCGGGGGTC |
| MLV U5 LTR Index Primer - GGCTAC | | *AATGATACGGCGACCACCGAGATCTACAC*TCTTTCCCTACACGACGCTCTTCCGATCT**GGCTAC**TGACTACCCGTCAGCGGGGGTC |
| MLV U5 LTR Index Primer - TAGCTT | | *AATGATACGGCGACCACCGAGATCTACAC*TCTTTCCCTACACGACGCTCTTCCGATCT**TAGCTT**TGACTACCCGTCAGCGGGGGTC |
| MLV U5 LTR Index primer - GTGGCC | | *AATGATACGGCGACCACCGAGATCTACAC*TCTTTCCCTACACGACGCTCTTCCGATCT**GTGGCC**TGACTACCCGTCAGCGGGGGTC |
| MLV U5 LTR Index primer- CGTACG | | *AATGATACGGCGACCACCGAGATCTACAC*TCTTTCCCTACACGACGCTCTTCCGATCT**CGTACG**TGACTACCCGTCAGCGGGGGTC |
| MLV U5 LTR Index primer - GAGTGG | | *AATGATACGGCGACCACCGAGATCTACAC*TCTTTCCCTACACGACGCTCTTCCGATCT**GAGTGG**TGACTACCCGTCAGCGGGGGTC |
| MLV U5 LTR Index primer - ACTGAT | | *AATGATACGGCGACCACCGAGATCTACAC*TCTTTCCCTACACGACGCTCTTCCGATCT**ACTGAT**TGACTACCCGTCAGCGGGGGTC |
| MLV U5 LTR Index primer - CTTGTA | | *AATGATACGGCGACCACCGAGATCTACAC*TCTTTCCCTACACGACGCTCTTCCGATCT**CTTGTA**TGACTACCCGTCAGCGGGGGTC |
| MLV U5 LTR Index primer - ATTCCT | | *AATGATACGGCGACCACCGAGATCTACAC*TCTTTCCCTACACGACGCTCTTCCGATCT**ATTCCT**TGACTACCCGTCAGCGGGGGTC |

^a^MLV U3 LTR second round PCR primers contain an adapter sequence (italics), primer binding sequence (underline), unique index/barcode (red) and U3 LTR sequence(plain black).

^b^MLV U5 LTR second round PCR primers contain an adapter sequence (italics), primer binding sequence (underline), unique index/barcode (red) and U5 LTR sequence(plain black).
