# Supplementary material for: Disrupting MLV integrase:BET protein interaction biases integration into quiescent chromatin and delays but does not eliminate tumor activation in a MYC/Runx2 mouse model
Source: PLoS Pathog. 2019 Dec 9;15(12):e1008154. doi: 10.1371/journal.ppat.1008154 (PMC6974304; doi:10.1371/journal.ppat.1008154)
Supplement: S8 Table — (DOCX) [file ppat.1008154.s013.docx]

**S8 Table. Genomic annotations and ChipSeq datasets used in the study*.***

| **Dataset** | **Accession number** | **Genome (reference build)** |
| --- | --- | --- |
| Brd4 | GSM1262345 | Mouse (mm8)^b^ |
| H3K27ac | ENCFF001KYZ | Mouse (mm10) |
| H3K4me1 | GSM1000102 | Mouse (mm9)^b^ |
| H3K4me3 | GSM1000101 | Mouse (mm9)^b^ |
| ChromHMM definition states | wgEncodeBroadHmmK562HMM | Human (hg18)^c^ |
| H3K27me3 | GSM733658 | Human (hg19) |
| H3K9me3 | GSM733776 | Human (hg19) |
| 9 state hi H3K27ac ^a^ | ENCSR032YTK | Human (hg19) |
| H3K36me3 | GSM733714 | Human (hg19) |
| H2A.Z | GSM733786 | Human (hg19) |
| H3K9me1 | GSM733777 | Human (hg19) |
| H3K9me3 | GSM733776 | Human (hg19) |
| H3K4me1 | GSM733692 | Human (hg19) |
| H3K79me2 | GSM733653 | Human (hg19) |
| H3K4me2 | GSM733651 | Human (hg19) |
| H3K4me3 | GSM733680 | Human (hg19) |
| Brd4 | GSM2635249 | Human (hg19) |
| CTCF | GSM733719 | Human (hg19) |
| H3K9ac | GSM733778 | Human (hg19) |
| ^a^Dataset was obtained from ENCODE, whereas other datasets were from NCBI Gene Expression Omnibus.  ^b^Coordinates based on prior genomic versions mm8 and mm9 were converted to mouse genome build mm10.  ^c^Coordinates based on prior genomic versions hg18 were converted to human genome build hg19. | | |
